# A Structural Model of the Human α7 Nicotinic Receptor in an Open Conformation

**DOI:** 10.1371/journal.pone.0133011

**Published:** 2015-07-24

**Authors:** Letizia Chiodo, Thérèse E. Malliavin, Luca Maragliano, Grazia Cottone, Giovanni Ciccotti

**Affiliations:** 1 Center for Life Nano Science @Sapienza, Istituto Italiano di Tecnologia, Rome, Italy; 2 Institut Pasteur and CNRS UMR 3528, Unité de Bioinformatique Structurale, Paris, France; 3 Center for Synaptic Neuroscience, Istituto Italiano di Tecnologia, Genoa, Italy; 4 Department of Physics and Chemistry, University of Palermo, Palermo, Italy; 5 School of Physics, University College Dublin, Dublin, Ireland; 6 Department of Physics, University of Roma “La Sapienza”, Rome, Italy; University of Saskatchewan, CANADA

## Abstract

Nicotinic acetylcholine receptors (nAchRs) are ligand-gated ion channels that regulate chemical transmission at the neuromuscular junction. Structural information is available at low resolution from open and closed forms of an eukaryotic receptor, and at high resolution from other members of the same structural family, two prokaryotic orthologs and an eukaryotic GluCl channel. Structures of human channels however are still lacking. Homology modeling and Molecular Dynamics simulations are valuable tools to predict structures of unknown proteins, however, for the case of human nAchRs, they have been unsuccessful in providing a stable open structure so far. This is due to different problems with the template structures: on one side the homology with prokaryotic species is too low, while on the other the open eukaryotic GluCl proved itself unstable in several MD studies and collapsed to a dehydrated, non-conductive conformation, even when bound to an agonist. Aim of this work is to obtain, by a mixing of state-of-the-art homology and simulation techniques, a plausible prediction of the structure (still unknown) of the open state of human α7 nAChR complexed with epibatidine, from which it is possible to start structural and functional test studies. To prevent channel closure we employ a restraint that keeps the transmembrane pore open, and obtain in this way a stable, hydrated conformation. To further validate this conformation, we run four long, unbiased simulations starting from configurations chosen at random along the restrained trajectory. The channel remains stable and hydrated over the whole runs. This allows to assess the stability of the putative open conformation over a cumulative time of 1 μs, 800 ns of which are of unbiased simulation. Mostly based on the analysis of pore hydration and size, we suggest that the obtained structure has reasonable chances to be (at least one of the possible) structures of the channel in the open conformation.

## Introduction

The nicotinic acetylcholine receptors (nAChRs), belonging to the Cys-loop super-family of ligand-gated ion channels (or LGICs), are membrane proteins present in neurons and at neuromuscular junctions [1]. They interact with a variety of ligands and are involved in various physiological processes [1–4] and pathologies [3, 5, 6]. The overall structure presents a fivefold symmetry, and it is composed of an extracellular domain (ECD, also called ligand binding domain, LBD, in eukaryotes), a transmembrane domain (TMD) and an intracellular part. The nAChRs comprise five subunits that can be identical or different and are arranged symmetrically to create the channel pore. Despite the large variability on the single components of the subunits, giving rise to the specificity of functions and location of these proteins, all subunits display the same architecture: the extracellular domain is composed mainly of *β* strands, while the transmembrane domain contains only *α* helices. The pore is formed by the alignment of one helix from each subunit, called M2. The orthosteric binding site of agonist and antagonist ligands is located in the LBD, and involves amino acid residues from two adjacent subunits, called principal and complementary. The C-loop, which is part of the principal subunit, caps the orthosteric binding site in the presence of a bound ligand. The nAChR channel pore, located in the TMD, opens following the binding of agonist ligands in the orthosteric site of the LBD. Conversely, the channel is mostly closed in the resting and in the inactive states, when either no ligand is present or an antagonist is bound, as well as in the desensitized, agonist-bound state.

Clearly, a detailed knowledge of the conformational transition and its dependence on ligand binding is critical to develop new pharmacological approaches influencing the receptor’s biological function. Unfortunately, despite enormous efforts devoted to their crystallization, as nAChRs are membrane proteins, little high-resolution information is available on their atomic structure [7] and on the gating mechanism. Partial knowledge is obtained from low resolution experimental data, from physiological studies coupled to mutagenesis [8] and/or from structures of proteins that share some functional or structural homology.

At present, insight on nAChRs atomic structure and mechanism comes from the following Cys-loop family members structures:

- two Electron Microscopy (EM) models of *Torpedo* acetylcholine receptor with closed [9] and open [10] pore;
- the X-ray crystallographic structures of two pH-gated prokaryotic homologues of nAChRs, one from *Erwinia chrysanthemi* (called ELIC) in closed form and one from *Gloeobacter violaceus* (called GLIC), initially assumed in open conformation [11–20].
- X-ray crystallographic structures of a water-soluble homologue of the LBD of nAChRs, the pentameric acetylcholine binding protein (AChBP), which has been co-crystallized with a number of agonists and antagonists [21–34]. The AChBP structures are the ones for which the best information on ligand-protein interactions within the orthosteric site is available for the moment [35–40]. Transferring conclusions to full-length channels, however, is not straightforward.
- the X-ray structures of some related eukaryotic proteins as the glutamate-gated chloride channel (GluCl) from *Caenorhabditis elegans* (open in presence of the allosteric agonist ivermectin [41] and in apo-closed conformation [42]), the human GABA_*A*_ receptor [43] (open) and the mouse 5HT3 receptor [44] (whose channel conductive state is not clear from experimental data).

Each of these structures displays structural and functional features which make them not fully relevant for the homology modeling of human nAChRs. However, the model obtained from the EM imaging of *Torpedo* acetylcholine receptor has been extensively used in the past for the first attempts of nAChRs homology modeling [45–50]. Normal mode analysis has been performed on top of coarse grain models of the protein. The normal mode of lowest frequency has been exploited to force the channel in the open state, both for homopentameric [45, 47, 48] and heteropentameric models of nAChRs [50], providing the first understanding of the conformational events leading to the channel opening. More recently, the X-ray structure of the eukaryotic GluCl raised hopes to be used as template for homology models of open channels. GluCl has been already used to model the full-length *α*1*β*2*γ*2 GABA receptor [51], and human glycine receptors bound to agonist and antagonist [52]. Unfortunately, MD simulations [53–55] pointed out an instability of the native GluCl structure which displays a tendency to collapse once the agonist ivermectin is removed [53, 55]. What is even worse, from another study [54] it appears that the presence of ivermectin barely affects the conformation of the TMD pore, which becomes dehydrated on a time scale of one microsecond.

As for the other eukaryotic structures listed above [43, 44], they are quite recent and no MD investigation on them has been made public up to now, to the best of our knowledge.

**Fig 1:**
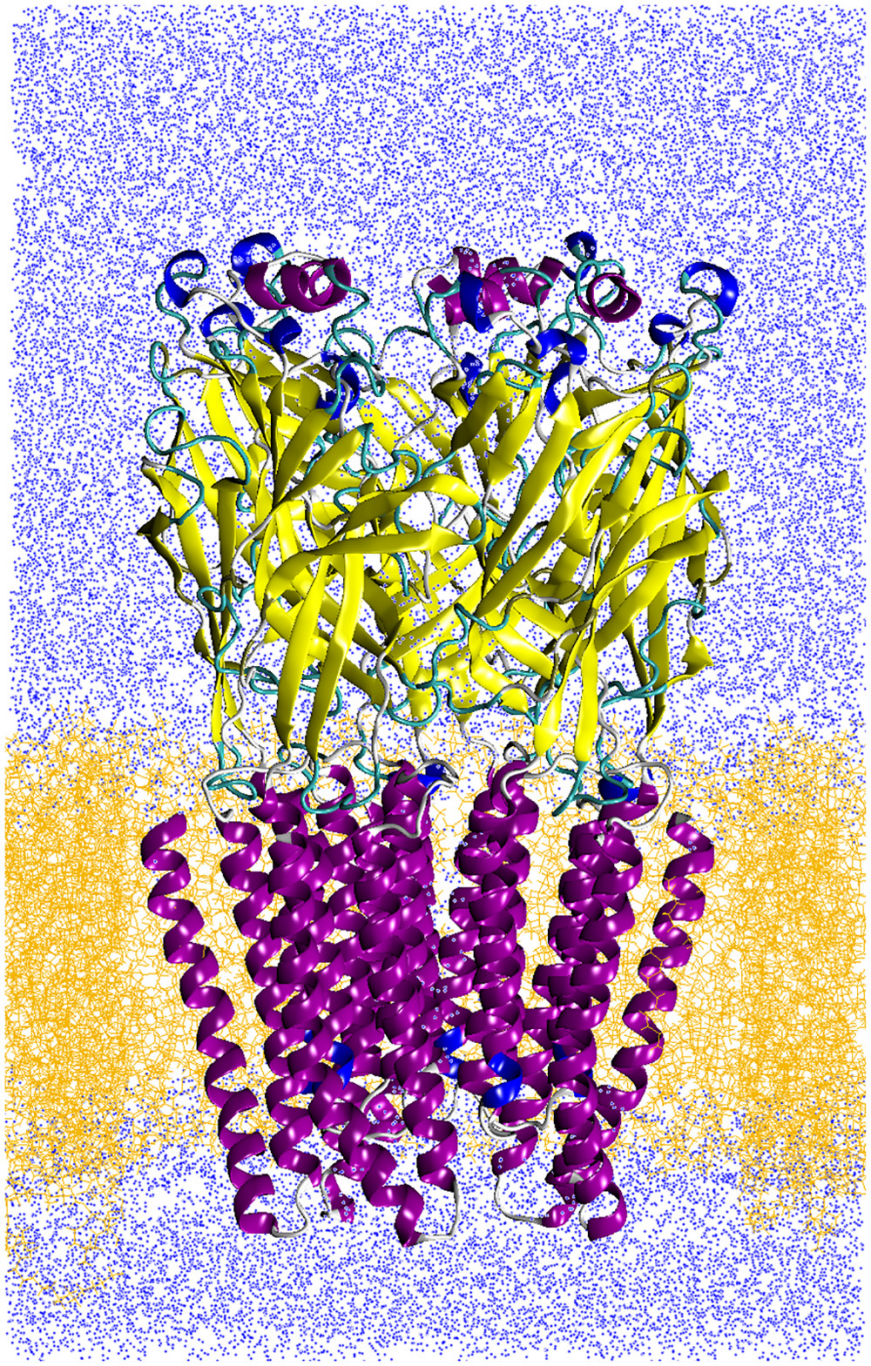
The system. Protein (in cartoon representation) embedded into fully solvated POPC lipids slab (yellow lines) and in physiological solvent (water, blue dots); Na and Cl ions and epibatidine ligands are not shown.

In the present work, we provide a first step towards obtaining an all-atom model of the human *α*7 full-length receptor in an open conformation (Fig 1), bound to the agonist epibatidine (S1 Fig). Our approach is based first on homology modeling of the channel using different high-resolution templates, and then on extended MD simulations of the protein structure solvated and embedded in a lipid bilayer. In order to obtain a consistent full structure in an open conformation we decided to build a chimera using as templates the agonist bound AChBP structure [26] for the LBD and the open GluCl structure [41] for the TMD. However, similarly to previous studies, in an unbiased simulation we observe a collapse of this structure toward a dehydrated conformation, even in presence of epibatidine. We then employ a restraint to avoid closure, and obtain in this way a stable hydrated conformation, which we eventually refine via extended, unbiased simulations.

The stability of the structures here obtained is assessed based on a cumulative simulation time of 1.2 *μ*s, 800 ns of which sample the unbiased open conformation. All structures were analyzed using several descriptors that have been already invoked to discriminate between open and closed channel conformations [50, 53–55], such as the pore profile, the water density inside the pore, the rigid-body motions of the TMD helices and the LBD/TMD quaternary twist. Analysis of the ion dynamics in the full-length channel, of the LBD-TMD interface and of the agonist conformations at the binding pockets contribute to the assessment of the obtained structures.

Consistent with previous findings [46, 50], our results show, in particular, that tilting of the helices and quaternary twist are not sufficient to properly describe the complex structural features of the channel. At variance, the analysis of the pore motif and hydration is much more informative: indeed, based on the features of the pore profile and the stationarity of the hydration level, we are able to discern two classes of stable structures from our simulations, and in particular to identify those representative of a possibly active state of the channel, which we refer to as putative open.

## Materials and Methods

### Homology modeling

**Fig 2:**
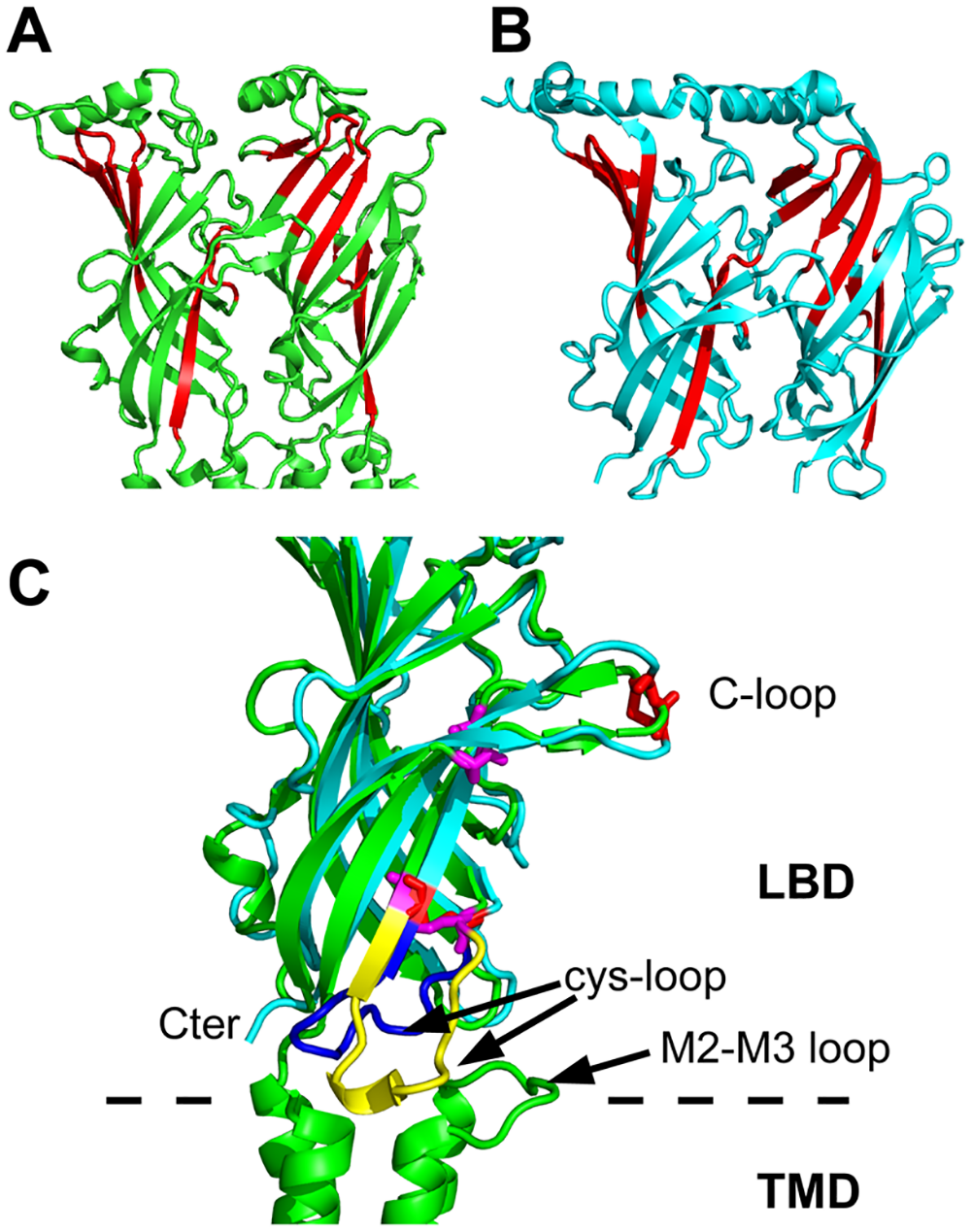
The chimera. A,B) X-ray crystallographic structures of the chains A and E of AChBP from *Aplysia californica* (PDB entry: 2BYQ [26]), and GluCl from *Caenorhabditis elegans* (PDB entry: 3RIF, [41]), respectively. The structures are drawn in cartoons, and the regions used for superimposition are colored in red. The residues of the superimposed structures are: (i) 55–62, 77–80, 107–118, 91–93, 138–147 for 2BYQ, and (ii) 54–61, 76–79, 110–121, 91–93, 141–150 for 3RIF. C) Superimposed subunits from 2BYQ (green) and 3RIF (in blue) using the residues defined above. The Cys-loop regions are colored in blue for 2BYQ and in yellow for 3RIF, and the cysteines of the Cys-loop are drawn in magenta for 2BYQ and in red for 3RIF. The superimposition and figure were realized using pymol [70].

**Fig 3:**
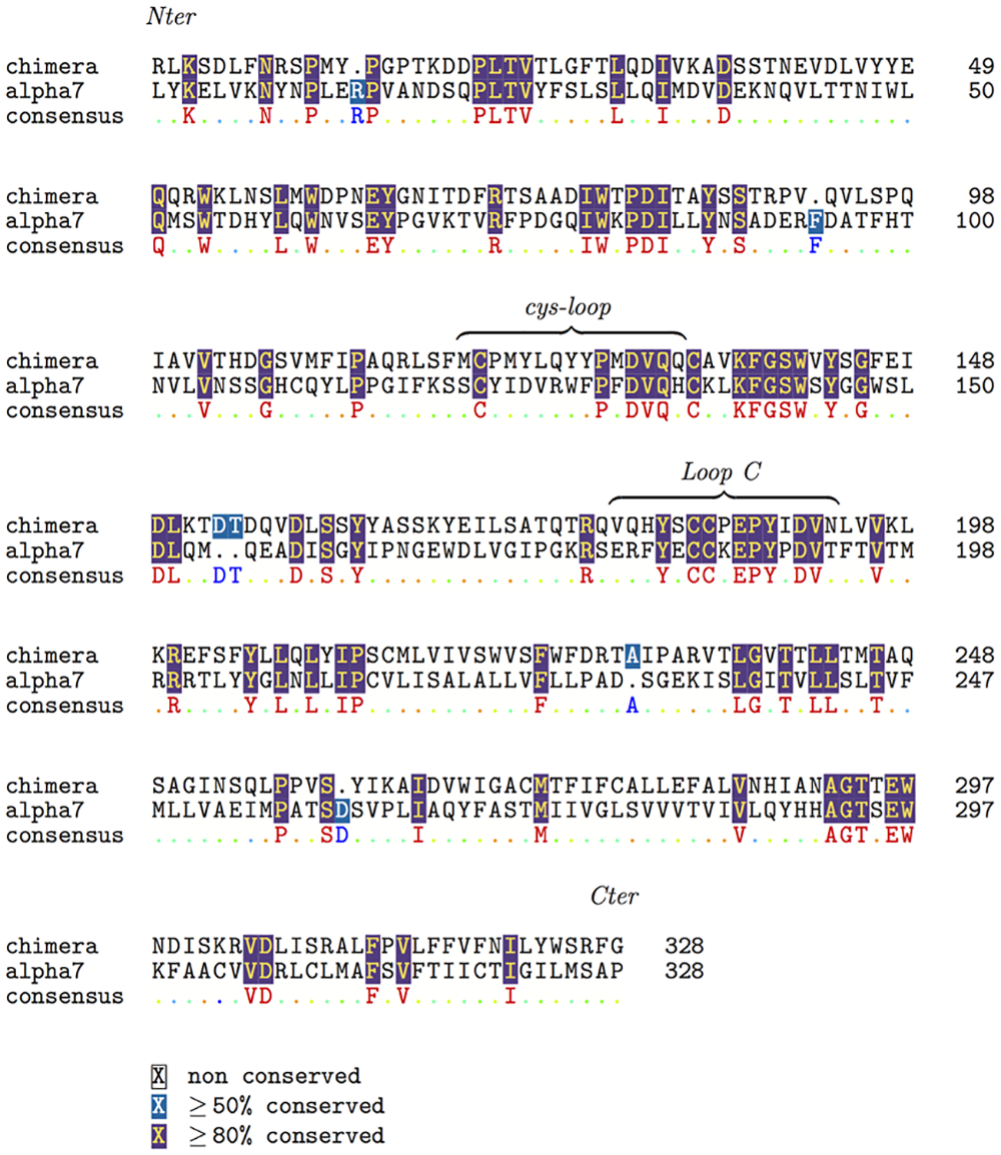
Sequence alignment. Alignment, produced by t_coffee 9.01 [64], of the *α*7 nAChR sequence after replacing the cytoplasmic region by a tripeptide linker and of the sequence of the chimera protein used as template. The N and C terminal regions of *α*7 nAChR have been removed due to poor alignments (data not shown) with other human *α* subunits of nAChR. The UniProt sequence P36544 was used for the *α*7 nAChR.

As templates for homology modeling of the *α*7 nAChR we used the following X-ray structures: (i) AChBP from *Aplysia californica* bound to epibatidine (PDB entry: 2BYQ [26]), (ii) GluCl from *Caenorhabditis elegans* (PDB entry: 3RIF [41]). The two template structures have been manually handled to build a chimera protein in which the ECD domain (residues 0–203 in the chain A of 2BYQ) except the Cys-loop (residues 127–140 in the chain A of 2BYQ) was taken from AChBP, and the TMD domain (residues 209–339 in the chain A of 3RIF) as well as the Cys-loop (residues 130–144 in the chain A of 3RIF) were taken from GluCl. The motivation for this cutting of the structures is given in Fig 2, where the chains A of 3RIF and 2BYQ are superimposed. Indeed, in the 3RIF structure, the Cys-loop (in yellow on Fig 2C) interacts with the M2-M3 loop of TMD, whereas the corresponding region in 2BYQ (in blue on Fig 2C) moves apart from the M2-M3 loop. As the reciprocal position of the Cys-loop and M2-M3 loop is known to play an important role in the conformational transition of LGICs [53, 56–59] we decided to use the chimera protein described below as a template for the homology modeling. The homology model of *α*7 nAChR was then built using the chimera template and a sequence alignment of *α*7 and the chimera. The percentage of identity between the two sequences is 25.37%, similarly to what obtained in previous works using the same template [60, 61]. Models built from this degree of identity have a Ca RMSD of 2-3 Å with respect to the true structure [62, 63]. In these cases, a proper alignment of the sequences before building the model becomes extremely important. The software t_coffee 9.01 [64] was used to align the full GluCl sequence, the truncated GluCl sequence present in 3RIF, the *α*7 nAChR, the sequences of human *α*2, *α*3 and *α*4 of nAChR, and the chimera sequences, to determine the cytoplasmic region of *α*7. This region is thought to be folded as an *α* helix from the models determined using the EM map recorded on *Torpedo* acetylcholine receptor [9]. As this region has been replaced by a tripeptide in the GluCl sequence used for the determination of the 3RIF structure, we decided to replace the equivalent region of the *α*7 nAChR by the same tripeptide. We also truncated the N and C terminal extremities which contained many insertions in sequences alignment. The alignment of the truncated *α*7 nAChR sequence along the chimera sequence is described in Fig 3. Our alignment shows only 6 gaps, while functional regions such as the C-loop, the Cys-loop, as well as many conserved residues, are perfectly matched. Results are in full agreement with the sequence alignment between 2BYQ and the LBD of *α*7 realized by Grazioso et al. [61] (compare Fig 1 in [61] with Fig 3). The homology modeling was realized using the script build_model.py from Modeller 9.10 [65], with the refinement protocol very_slow and 10 refined models.

The best homology model was determined as the one showing the smallest percentage of residues in the generously allowed and disallowed region of the Ramachandran diagram, calculated using Procheck 3.5.4 [66]. The model was analyzed by the Molprobity server [67]molprobity.biochem.duke.edu: (i) to add the hydrogens, and (ii) to flip residues in order to optimize the sidechain interactions. The Ramachandran plot of the model output of Molprobity [68] contained 16 outliers, all located in structure loops. The model was then analyzed using the QMEAN server swissmodel.expasy.org/qmean [69], obtaining a QMEAN norm score of 0.5. This score value is satisfying as it is only slightly smaller than the corresponding score values of 0.62 and 0.53 obtained respectively on the crystallographic X-ray structures 2BYQ and 3RIF which were the initial points for the template building.

### System set-up and Molecular Dynamics simulations

Protonation state of ionizable residues at physiological pH (7.4) was predicted by using the web-based implementation of a method for pKa calculations based on continuum electrostatic model [71] (http://biophysics.cs.vt.edu/H++[72]).

MD Simulations were performed with the NAMD2.7b2 package [73, 74], with the CHARMM27 force field for proteins, ions, and water and the CHARMM36 force field for phospholipids. Topology for the epibatidine (S1 Fig) was generated by Antechamber [75]; ligand partial atomic charges were obtained by us from *ab initio* calculations with Gaussian 03 [76]. Epibatidine parameters are given as Supplementary material, see file S1 Text. The protein has been inserted into a pre-equilibrated 110 × 110 Å lipid bilayer originally composed by 356 palmitoyl-2-oleoyl-sn-glycerol-phosphatidylcholine (POPC) lipids and 5605 waters molecules, obtained from CHARMM-GUI at www.charmm-gui.org. The protein-lipid system underwent 10000 steps of conjugate gradient minimization; the temperature of the system was then increased gradually to 300 K over 2.4 ns MD simulation, by a sequence of 200 ps long simulations at constant pressure (1 atm) and temperature, each with an increase of 25 K and with random reassignment of the atomic velocities at each temperature increase, to let the membrane equilibrate around the protein. At this stage, harmonic positional restraints were applied to all protein atoms, with a force constant of 5 kcal/mol/Å^2^. Water was then added to fully solvate the protein/lipid system; Na^+^ and Cl^−^ ions, corresponding to 100 mM solution were added to neutralize the net system charge. The total number of atoms is 142720 (26313 protein atoms, 27360 water molecules, 255 POPC lipids, 157 ions), see Fig 1. The system was again minimized for 10000 steps; harmonic positional restraints were applied to all lipid and protein atoms with a force constant of 5kcal/mol/Å^2^. The temperature of the system was then increased gradually to 300 K over 0.6 ns MD simulation, by a sequence of 100ps long simulations at constant pressure (1 atm) and temperature, each with an increase of 50 K and with random reassignment of the atomic velocities at each temperature increase, to let the water and the ions equilibrate around the protein and the membrane. Further 5 ns were then run in the NPT ensemble at 300 K and 1 atm, by gradually removing the positional restraints on all protein atoms (force constant from 5 to 1 kcal/mol/Å^2^, 1 ns for each force constant value), this time allowing lipids, water and ions to move freely.

Periodic boundary conditions were applied, with particle-mesh Ewald long-range electrostatics [77], using a grid spacing of 1 Å along with a sixth order B-spline charge interpolation scheme. A cutoff of 1.2 nm for Lennard-Jones potential, with a smooth switching function starting at 1.0 nm, was used. Bonds involving hydrogen atoms were constrained to their equilibrium length using the SHAKE/RATTLE algorithm [78], with time step of 1 fs.

We first ran two different simulations, using as starting condition the last conformation from the equilibration trajectory (see Table 1). In the first simulation, which we call *unrestrained*, the system is simulated for 200 ns in the NPT at 310 K and 1 atm with the protein unrestrained. We anticipate here that over the first few ps of this trajectory we observe a collapse of the pore channel toward a non-hydrated structure. The implications are thoroughly discussed in the Results section. This provided us with what we call in the following the *collapsed* channel, and prompted us to perform a second simulation, which we call *restrained*. The second simulation was run for 200 ns in the NPT at 310 K and 1 atm; with the aim at obtaining an open conformation, in this run a restraint has been applied on the two hydrophobic rings made of Leucine and Valine residues in position 16′ and 13′ that constitute the constriction point of the pore channel. We designed a “flat-bottom quadrati” potential acting on the distances between the centers of mass of Leu16′ and Val13′ on pairs of non-adjacent subunits [79]. The potential is a function of the distance that discourages values smaller than a threshold via a quadratic expression, and it is zero otherwise. The threshold value corresponds to the initial (open) structure; a force constant of 10 kcal/mol/Å^2^ has been used. Also the restrained simulation was run with NAMD, the restraint being implemented via Tcl routines linked in the NAMD code itself. Time series of the restrained distances are shown in S2 Fig. The analysis of their characteristics described below, suggest that the conformations obtained in this trajectory are representative of the channel structure in an open form.

**Table 1:** Simulations of the *α*7 pentamer.

Name	Starting point	Components	Duration (ns)
unrestrained	equilibrated homology model	*α*7 channel, EPI	200
restrained	equilibrated homology model	*α*7 channel, EPI	200
Free_1_	restrained trajectory at 20ns	*α*7 channel, EPI	200
Free_2_	restrained trajectory at 31ns	*α*7 channel, EPI	200
Free_3_	restrained trajectory at 34ns	*α*7 channel, EPI	200
Free_4_	restrained trajectory at 70ns	*α*7 channel, EPI	200

Details of the MD trajectories. EPI: abbreviation for Epibatidine.

Four more simulations, in the following called *Free_1_,…, Free_4_* were also run in the NPT at 310 K and 1 atm, each one for 200 ns (see Table 1), starting from four initial configurations picked along the restrained trajectory at times where the restraint was not active. The similarity of the three-dimensional structures of these four initial configurations was assessed by measuring the Root Mean Square Distance of all *C*_*α*_ atoms, after optimal rigid body superposition of pairs of structures (i.e. *Free_*i*_ vs Free_*j*_*, i,j = 1, 4). The RMSDs values are in the range 1.3 Å - 2.2 Å, pointing out they are representative of the same conformation. No restraint has been applied along these four trajectories.

Summarizing, we have three sets of trajectories (*unrestrained*, *restrained* and *Free_1,2,3,4_*), which provided us with two classes of conformations: the collapsed channel (from the *unrestrained* trajectory) and the putative open channel (from the *restrained* and *Free_1,2,3,4_* trajectories). Therefore, the stability of the putative open state here presented has been assessed over a cumulative simulation time of about 1.0 *μ*s, 800 ns of which are unbiased.

### Trajectory analysis

To assess the overall stability of the new homology model, we calculated: i) the *C*_*α*_ atoms Root Mean Square Deviation (RMSD), from the configuration at the end of the equilibration stage, separately for the LBD and the TMD of the five subunits (in the following called P_1_,…, P_5_); ii) the *C*_*α*_ atoms Root Mean Square Fluctuations (RMSFs) with respect to the average positions calculated over the final 100 ns of each trajectory.

**Fig 4:**
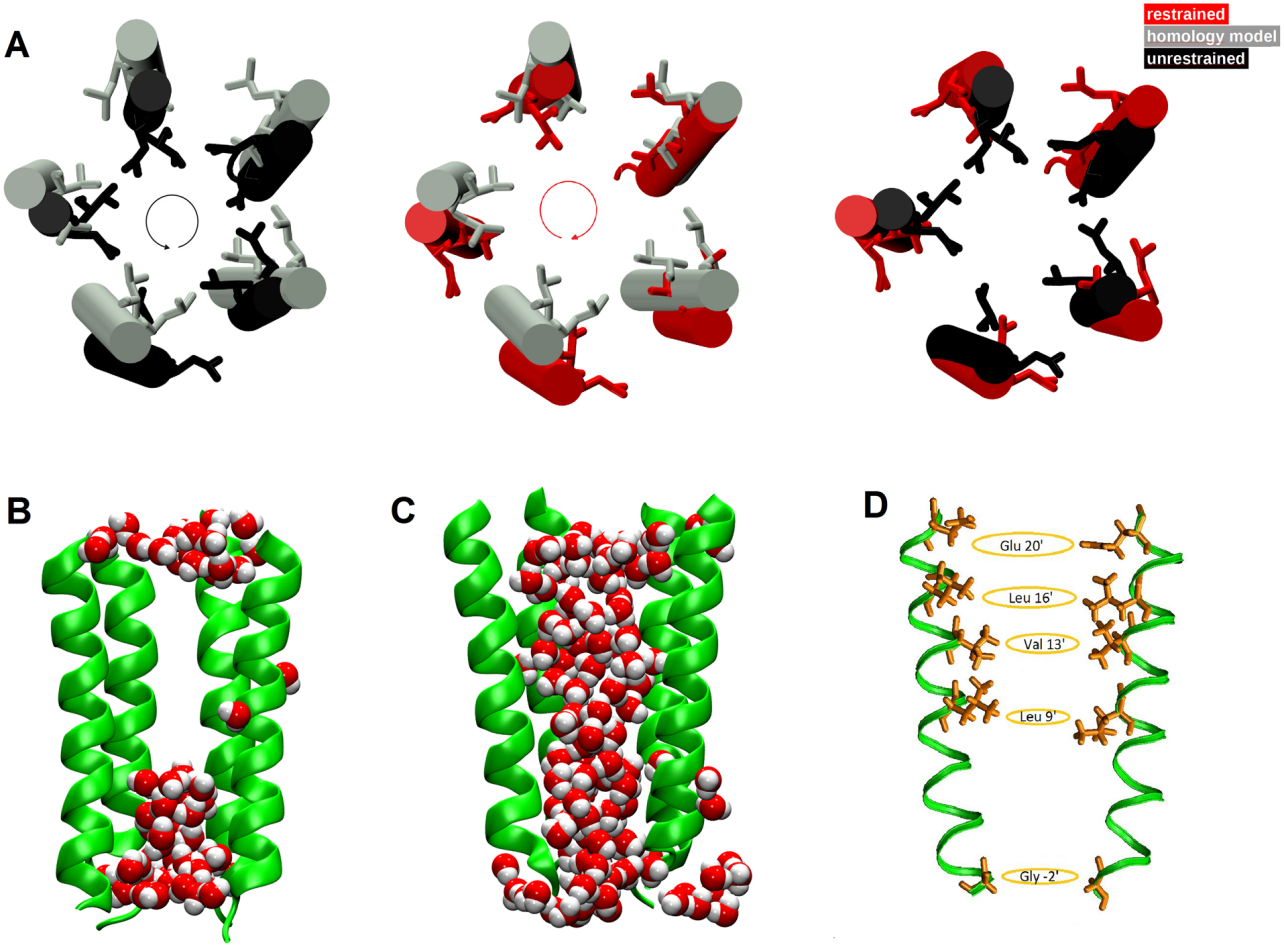
Pore structure and hydration. A) Top view of M2 helices and orientation of the pore-delimiting hydrophobic side chains in the TMD of the collapsed channel (black) and open channel (red) in the structures averaged over the last 100ns of the unrestrained and restrained trajectories, respectively. The initial homology model structure is in grey. The pore size in the open channel enlarges due to side chains swinging away from the center of the pore (see e.g. in Ref. [50]). B) Snapshots of the M2 helices in the collapsed channel; C) in the putative open channel along the restrained simulation. The M2 helix of the subunit in the front is removed to reveal the water molecules inside the pore, in vdW representation. D) Hydrophobic (Leu16′, Val13′, Leu9′) and hydrophilic (Glu20′, Gly2′) residues rings in the pore. The crossed distances between pairs of non-adjacent subunits for Leu at 16′ and Val at 13′ have been restrained with a flat-bottom potential to drive the pore towards the open state.

To describe the channel pore we calculated along all trajectories: i) the distances between specific residues of the M2 helices in non-adjacent subunits (crossed distances); ii) the time evolution of the number of water molecules in selected regions (the full pore channel, as enclosed by the five M2 helices between Gly2′ and Glu20′, 30 Å long; the constriction point between Leu9′ and Leu16′, 10 Å long, Fig 4D); iii) the pore radius, computed using the web-based implementation of a method to compute molecular channels [80] (http://bioinfo3d.cs.tau.ac.il/MolAxis/) in the configuration obtained by averaging over the last 100 ns portion of each trajectory; iv) the orientation of the five M2 helices and the quaternary twist angle between the LBD and TMD of each subunit [53].

Given that the M2 helices in the *α*7 do not undergo bending, we describe their orientation in terms of the tilt angle that the overall helix (*C*_*α*_ atoms of 23 residues, from Gly2′ to Glu20′) forms with the protein axis. The comparison of the available X-ray structures of the putative closed and open forms of nicotinic receptors points out that in the opening/closure conformational transition the M2 helices undergo both a radial tilting and a tangential motion. For this reason, the M2 helix tilt has been usually decomposed into polar and azimuthal components [48]. As in Ref. [53], we use a reference frame centered on the center of mass of the M2 helix, with the z axis parallel to the protein symmetry axis and the x axis pointing outward along the radial direction. The polar tilt is then the angle between the z axis and the projection of the helix axis on the xz plane. The azimuthal tilt is the angle between the x axis and the projection of the helical axis on the xy plane. The twist angle is calculated as the angle between the projections of the vectors from the whole protein center of mass to the center of mass of the LBD and TMD respectively (only the *C*_*α*_ atoms are considered), on the plane perpendicular to the symmetry axis of the protein.

Quantities assessing the LBD and the LBD-TMD interface were analyzed: the C-loop/backwall distance ($d_{C}^{intra}$), defined as the distance between the center of mass of the C-loop (*C*_*α*_ atoms of residues 179–188), capping the epibatidine molecules, and the center of mass of *C*_*α*_ atoms of residues 139–140, in the backwall [40]; ligand-protein distances; intra-protein hydrogen bonds; the M2-M3 loop distance with respect to the Cys-loop and the *β*1-*β*2 loop, respectively, at the LBD-TMD interface. Distributions of cations and anions in the LBD region and TMD channel have been calculated as well.

## Results

In the following sections we discuss the stability of our model, and we assess its conformation via comparative inspection of pore geometry, hydration behavior, the LBD-TMD twist angle and the M2 helices orientation. The dynamics of ions within the channel is analyzed as well. Finally, the pattern of intra-protein hydrogen bonds and the interaction of the agonist ligands with the LBD are discussed. We compare data obtained in the three sets of trajectories described in the Methods section (*restrained*, *unrestrained* and *Free_1,2,3,4_*). Results suggest that conformations extracted from these trajectories are representative of the collapsed and putative open forms of the channel.

### Model stability assessment

In this section we report mostly on the analysis of the unrestrained and restrained simulations, performed to probe the stability of the homology model and based on RMSD and RMSFs calculations (S3 Fig). The time behavior of the C*α* RMSD over 200ns indicates that the conformation of the channel bound to epibatidine is stable on this time scale, both at the LBD and TMD level. The overall drift is slightly more pronounced (by 0.5 Å) in the restrained simulation than in the unrestrained one, remaining however below 4 Å in the LBD domain and below 3 Å in the TMD domain. The time behavior of the C*α* RMSD over the restrained trajectory, calculated with respect to the average collapsed conformation (data not shown), is stationary around 3.3 Å for the full channel, 2.7 Å for the LBD and 1.9 Å for the TMD (averaged over the five subunits), indicating that the two conformations are globally different. Local differences are extensively discussed below.

The RMSFs along protein sequence is similar to the one obtained from previous simulations of homology models of human receptors based on different protein templates [45]. For example, in both simulations we observe a large peak in the region of helix M4 around residue 300. The high flexibility of this region is a known consequence of the structural uncertainty associated to the loop M3-M4 [81]. From a comparison of the RMSFs in the restrained and unrestrained trajectories, we clearly see no differences in peak positions, but differences in peak amplitudes. In particular, the A-loop and the F-loop are more mobile along the restrained trajectory. Otherwise, the C-loop, that caps the epibatidine molecule, and the Cys-loop, that drives the interaction with the TMD, show similar behavior in the two systems.

As for free trajectories, the time behavior of *C*_*α*_ RMSDs with respect the initial condition and the *C*_*α*_ RMSFs are both reported in Supplemental Material (S4 Fig). Results point out a substantial stability of the structures along the four free trajectories; the overall RMSD drift is less pronounced than in the unrestrained simulation (S3 Fig). Together with the observation that the four initial conditions are representative of the same putative open form (see “System set-up and Molecular Dynamics simulation”), this allow the assessment of the putative open channel at least on a cumulative time of 800 ns.

### Pore geometry and hydration

#### Unrestrained trajectory

**Fig 5:**
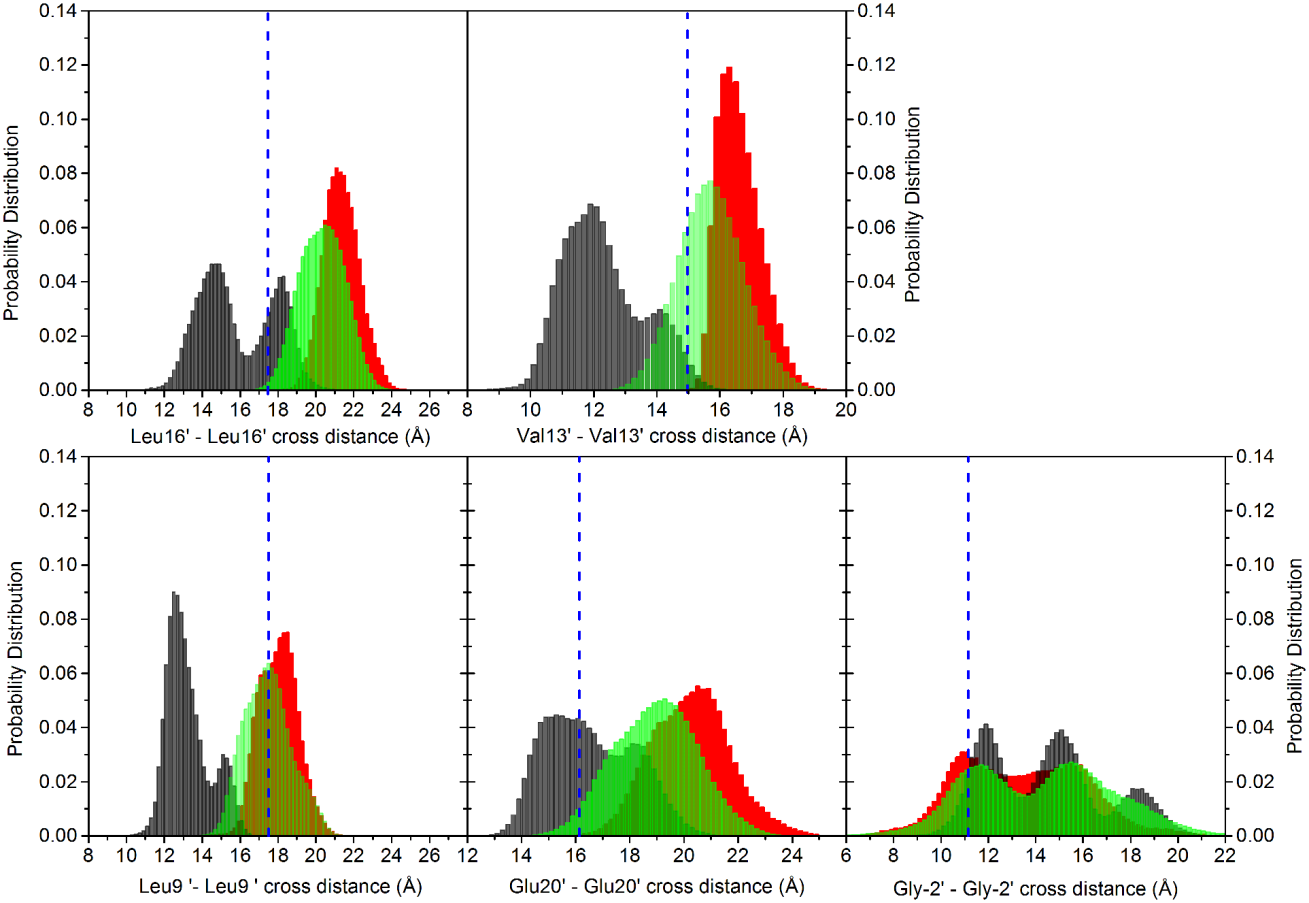
Distribution of the crossed distances. Distributions of crossed distances between pairs of non-adjacent subunits for Leu at 16′, Val at 13′, Leu at 9′, Glu at 20′ and Gly at -2′; data collected from the five subunits. The vertical dashed blue line represents the value in the starting homology modeled configuration. Black bars: unrestrained; red bars: restrained; green bars: free simulations (data collected from the four free trajectories).

In this simulation, a spontaneous conformational change is observed at the equilibration stage in few ps, as soon as the protein positional restraints are removed, and it is irreversible over the following 200 ns time scale of the MD trajectory. This change is characterized by a sudden decrease of the inter-residues crossed distances between M2 helices (S5 Fig, dotted lines). The drop in crossed distances is a consequence of an overall anticlockwise rigid movement of the M2 helices, which causes rotation of sidechains of hydrophobic residues Leu9′, Val13′, Leu16′ towards the inner part of the channel (Fig 4A). Distributions of the distances between couples of residues pertaining to the same hydrophobic ring are shown in Fig 5.

The distributions were determined by merging the time series of the distances collected for the individual pairs; this to include the contributions of conformational transitions from individual subunits that would be otherwise blurred by averaging. It is evident from the multimodal distributions that different subunits can undergo slightly different behaviors, highlighting an intrinsic asymmetric behavior of the pentameric protein [45, 82]. The value obtained for our homology model is also reported as reference (Fig 5, blue dashed vertical line). In the collapsed channel (Fig 5, black bars), the Leu16′, Val13′, Leu9′, Glu20′ rings undergo a contraction, while the Gly2′ ring delimiting the intracellular part of the pore undergoes a widening with respect to the starting structure.

**Fig 6:**
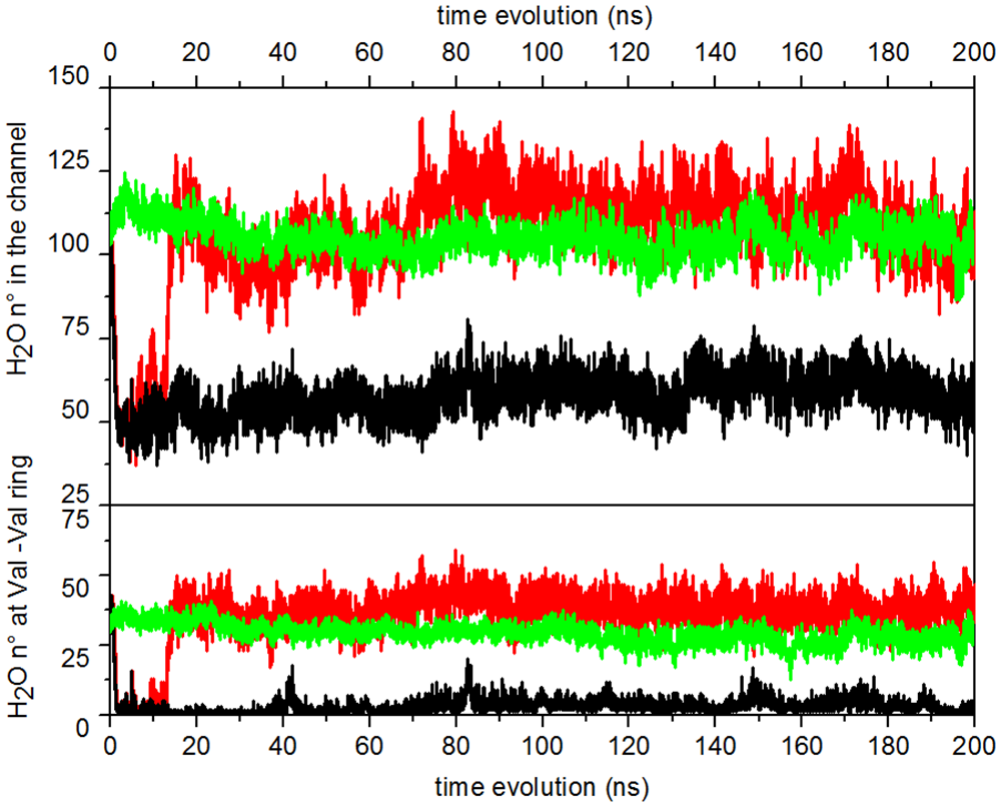
Hydration behavior. Upper panel: time evolution of water count in the pore lumen lined by the M2 helices. Lower panel: time evolution of water count in a region of 10 Å centered at the constriction point 13′ (Val246). Black curve: unrestrained; red curve: restrained; green curve: free trajectories (data averaged over the four free trajectories).

This new configuration creates a hydrophobic steric hindrance that inhibits water passage, and therefore the ion permeation along the channel. Fig 6 shows the time series of the number of water molecules inside the channel pore. The number of waters in the full channel (30 Å length) is stationary around 60 over the full simulation (Fig 6, upper panel, black curve). The number of waters in the region of Leu9′-Leu16′ (10 Å length) is of the order of few molecules (4 on average), with (unsuccessful) attempts of rewetting [83] at ∼ 5ns, ∼ 42ns, ∼ 82ns, ∼ 117ns, ∼ 150ns, ∼ 175ns and ∼ 190ns along the 200 ns trajectory (Fig 6, lower panel, black curve). A representative snapshot of the M2 helices and water inside the pore in the collapsed form is shown in Fig 4B.

#### Restrained trajectory

The restraint applied on the crossed distances in the rings formed by Leu16′ and Val13′ residues induces a completely different hydration state in the channel. Distributions of the crossed distances are shown in Fig 5, red bars. Their time evolution, over the first 20ns of the trajectory, is shown in S5 Fig (solid lines). Remarkably, the restraint has non-local structural consequences: the crossed distances at the rings Glu20′, Leu9′, and Gly2′, not affected by it, show also a structural change (Fig 5, red bars). In particular the Glu20′ and Leu9′ rings undergo an increase, while the Gly2′ ring slightly shrinks. This suggests that the distance restraints induce a collective motion of the M2 helices. Indeed, as shown in Fig 4A, the M2 helices undergo a clockwise rotation with respect to the initial structure, with the hydrophobic sidechains pointing outside the pore.

As already observed in a study of channel opening of models of *α*7 based on normal mode analysis [47], the M2 rotation cause the pore to open up, allowing the water passage. In fact, as shown in Fig 6, the number of waters grows in almost 10ns, following the widening of the V-ring and L-ring due to the applied restraint. After this transition, the overall filling of the channel is almost doubled, becoming stable at around 115 water molecules (Fig 6, upper panel, red curve). Also the hydrophobic gate is now completely hydrated, with almost 40 molecules in the region corresponding to the constriction point (Fig 6, lower panel, red curve). A representative snapshot of the M2 helices and water inside the pore in this putative open form is shown in Fig 4C.

#### Free trajectories

Our most important result is that the restraint applied to the crossed distances between residues of the hydrophobic gate induced a reorganization of residues positions and, as a consequence, a new network of interactions capable of generating a conformation that is quite different from the collapsed one. To further validate and refine this conformation we submitted it to four independent, 200 ns long, free MD simulations.

In particular, the channel hydration observed in the restrained simulation is retained along the free trajectories (S6 Fig). The time evolution of the number of water molecules, averaged over the four trajectories, is shown in Fig 6 (green curve). Distributions of the crossed distances averaged over the four free trajectories show profiles similar to the one in the restrained trajectory (Fig 5, green bars).

Hydrations relaxes at stable values slightly lower than in the restrained simulation (95 waters in the full channel, 30 at the hydrophobic girdle), although still much higher than in the collapsed channel, both in the whole channel and at the hydrophobic gate. Events of sudden dewetting followed by rewetting are observed in one of the four trajectories (S6 Fig, violet curve, at ∼ 115 ns, ∼ 135 ns, ∼ 145 ns, ∼ 180 ns).

The observation that water filling slowly follows channel opening after restraint application (10ns at least) suggests that the water pressure is not the primary cause for opening the pore, but instead it is a (meta-)stable conformation induced by the presence of the restraint, allowing water filling but also physiological dewetting-rewetting events. Notably, the same initial structure was used for both the restrained and unrestrained simulations, built using as template for the TMD the allegedly open GluCl structure (3RIF). After the equilibration stage, the number of water molecules in this structure is ∼ 100 in the full channel and ∼ 40 in the hydrophobic gate region. If water pressure was the primary factor contributing to the stabilization of the pore in an open form, then we should have not observed pore collapse in the simulation without restraints on protein atoms.

**Fig 7:**
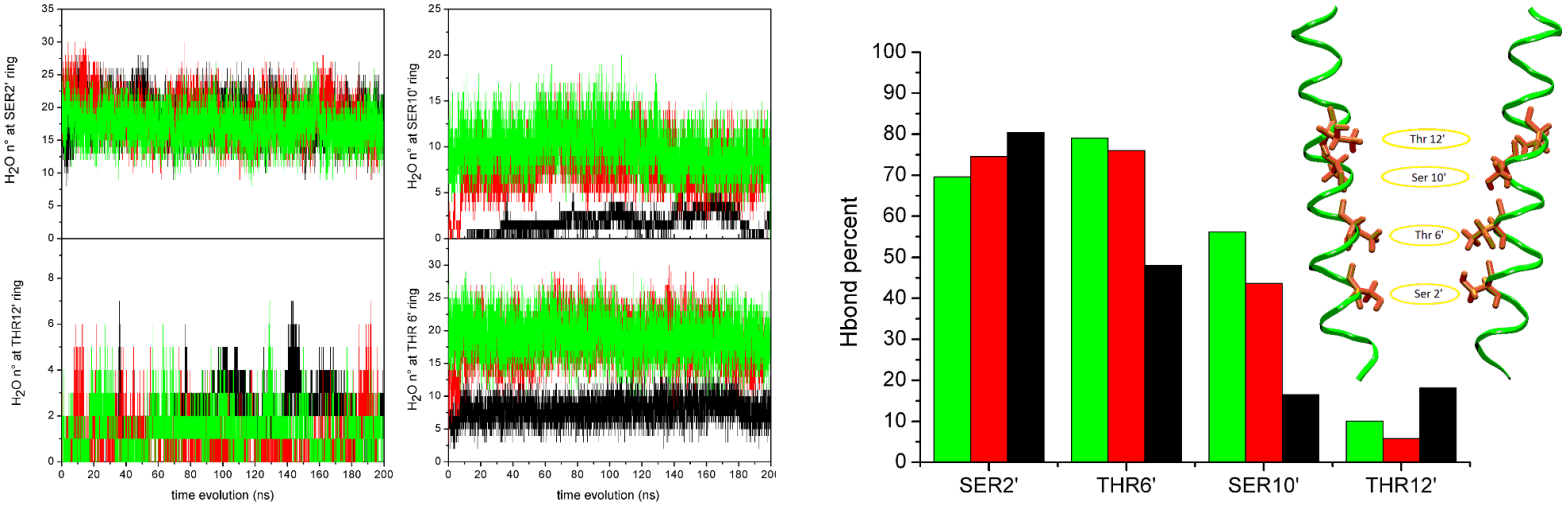
Hydration behavior. Left and central panels: time evolution of water count within 3 Å from any atom of Ser2′, Ser10′, Thr6′ and Thr12′ in the pore channel. Black curves: unrestrained; red curves: restrained; green curve: trajectory Free_4_. Right panel: percentages of of hydrogen bond formation between water molecules and protein residues along the trajectories. Black bars: unrestrained; red bars: restrained; green bars: trajectory Free_4_. The hydrogen bond is considered to be formed when the minimum distance among all possible donor/acceptor distances, between the two partners, is less than 2Å.

To investigate a possible role of water in stabilizing the pore from collapse successive to the effect of the restraint, we analyzed the water dynamics at the level of the hydrophilic rings in the pore, where a significant and persistent water population is expected. First, the number of water molecules found within 3 Å from any atom of Ser2′, Ser10′, Thr6′ and Thr12′ rings has been counted along the unrestrained, restrained and the Free_4_ trajectory, selected as representative of the four free trajectories in which the restraints were not applied. The time behavior of the selected water count is shown in Fig 7, left and central panels. In addition, residue-water hydrogen bond analysis has been performed. The hydrogen bond is considered to be formed when the minimum distance among all possible donor/acceptor distances, between water and protein residue, is less than 2.0Å. The persistence of the selected hydrogen bond along the simulations, defined as the percentage of time along a full trajectory that the bond is formed, is also shown in Fig 7, right panel (data averaged over the five subunits).

Results show that the number of water molecules surrounding the Ser10′ and Thr6′ rings discriminates among the collapsed and putative open protein conformations, while similar behavior among the restrained, unrestrained and free trajectory is observed for water at Ser2′ (located at the channel bottom) and at Thr12′. In particular, the number of water molecules in the Ser10′ domain significantly increases when the pore is opened by the restraints (Fig 7, red curve) with respect to the collapsed trajectory (Fig 7, black curve); the water filling slowly follows channel opening (∼ 10ns), as already observed in the time series of the total count of water in the pore (see Fig 6). The filling is likely induced by the enlargement of the Leu9′ ring, a consequence of the presence of the restraint (see above, “Restrained trajectory”). The number of water at Ser10′ is stationary in the free trajectory, after the restraints are turned off (Fig 7, green curve). Representative snapshots of water molecules inside the pore at Ser10′ and Thr 6′ respectively, taken at different times along the Free_4_ trajectory, are shown in S7 Fig.

Water/residue hydrogen bonds are persistently present along the restrained and free trajectories to a greater extent than in the collapsed one.

By looking at the time behavior of the number of water molecules in the pore in the various trajectories (Fig 6), we first observe a sudden dewetting at the early steps of both the restrained and unrestrained trajectories (t < 10ns). In the former however, between 10 and 20ns water is able to re-enter and stably hydrate the pore. From a careful inspection of the trajectories it emerges that this event is favored by the presence of the restraint, which creates accessible volume in the pore by pushing apart the sidechains of the hydrophobic residues. Once inside the pore, the water molecules settle in a (meta-)stable conformation (see e.g. water rings in S7 Fig) and actively contribute to keep the pore open even when the restraint is off (portions of restrained trajectory and free trajectories), by preventing the residues to change conformation and in particular the hydrophobic residues to rotate back towards the pore and consequently the helices to collapse.

### Pore profile

**Fig 8:**
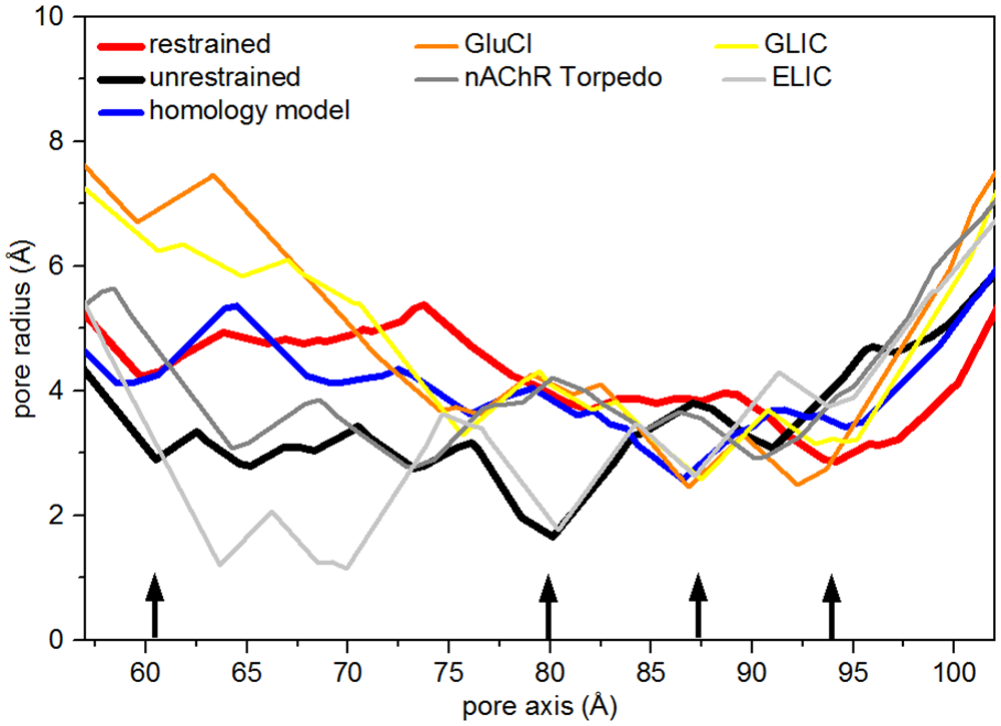
Pore profile. Pore radius profiles along the pore region in the homology model configuration (blue curve), in the collapsed (black) and open (red) structures averaged over the last 100ns of the unrestrained and restrained trajectories, respectively. Also reported the profiles for open channels (GluCl and GLIC from X-rays) and closed channels (Torpedo nAChR from EM and ELIC from X-rays). Pore radius computed with MolAxis [80]. MolAxis is a channel finding algorithm, in which molecular channels are represented using “corridors”, i.e. probable routes taken by a small molecule passing through a channel. MolAxis uses an algorithm that allows fast identification of corridors in the complementary space of the molecule. The black arrows, from left to right, indicate Glu20′, Leu9′, Thr6′ and Gly2′.

Results so far reported depict two different situations in the collapsed and putative open forms (both from the restrained and the free trajectories), in what concerns the pore geometry and the consequent hydration behavior. Fig 8 shows the pore radius along the channel axis (region corresponding to the M2 helices). We compare our homology model, the collapsed channel and the putative open channel obtained from the restrained simulation, together with the profiles from the structures of GluCl [41] (X-rays, open in the presence of ivermectin), GLIC [15] (X-rays, open), ELIC [17] (X-rays, closed), and Torpedo nAChR [9] (EM, closed). The pore profiles calculated in the free trajectories are shown in S8 Fig. The collapsed structure has one constriction point, corresponding to the Leu9′ rings, with a minimum radius of ∼ 2 Å. This constriction point at Leu9′ is in common with the ELIC profile. It has been also observed in the relaxed native GluCL both in the presence and in the absence of ivermectin [54], and in a model of a Glycine receptor built on GLIC [84] (radius equal to 1.4 Å at 9′), both in the presence or absence of ethanol, included in the simulation with the aim to stabilize the Glycine receptor in an open form.

On the other hand, the radius of our collapsed channel is about 3 Å in the hydrophobic gate. The putative open channel has instead a quite large radius (∼ 5 Å, comparable to the ∼ 5 Å found in GLIC [15]) at the hydrophobic gate, allowing water permeation, and a constriction point of radius ∼ 3 Å at the intracellular entrance (in correspondence to Gly2′, near the selectivity filter (Glu1′)).

For comparison, closed and putative open channel models of human *α*4*β*2 nAChR obtained by homology modeling and MD simulations [50] have radii of 2.7 Å and 3.4 Å at the extracellular entrance (corresponding to our Glu20′), respectively. A constriction point, observed for the homology model at Thr6′ (Fig 8, blue curve), is present in GluCl, GLIC and ELIC, whereas it has disappeared in the putative open channel (red curve). The collapse of the homology model might be induced by the presence of this constriction point, whereas the action of the restraints induces the opening of the Thr6′ ring and the consequent water filling already observed in Fig 7.

### Large scale motions: quaternary twist and M2 orientation

**Fig 9:**
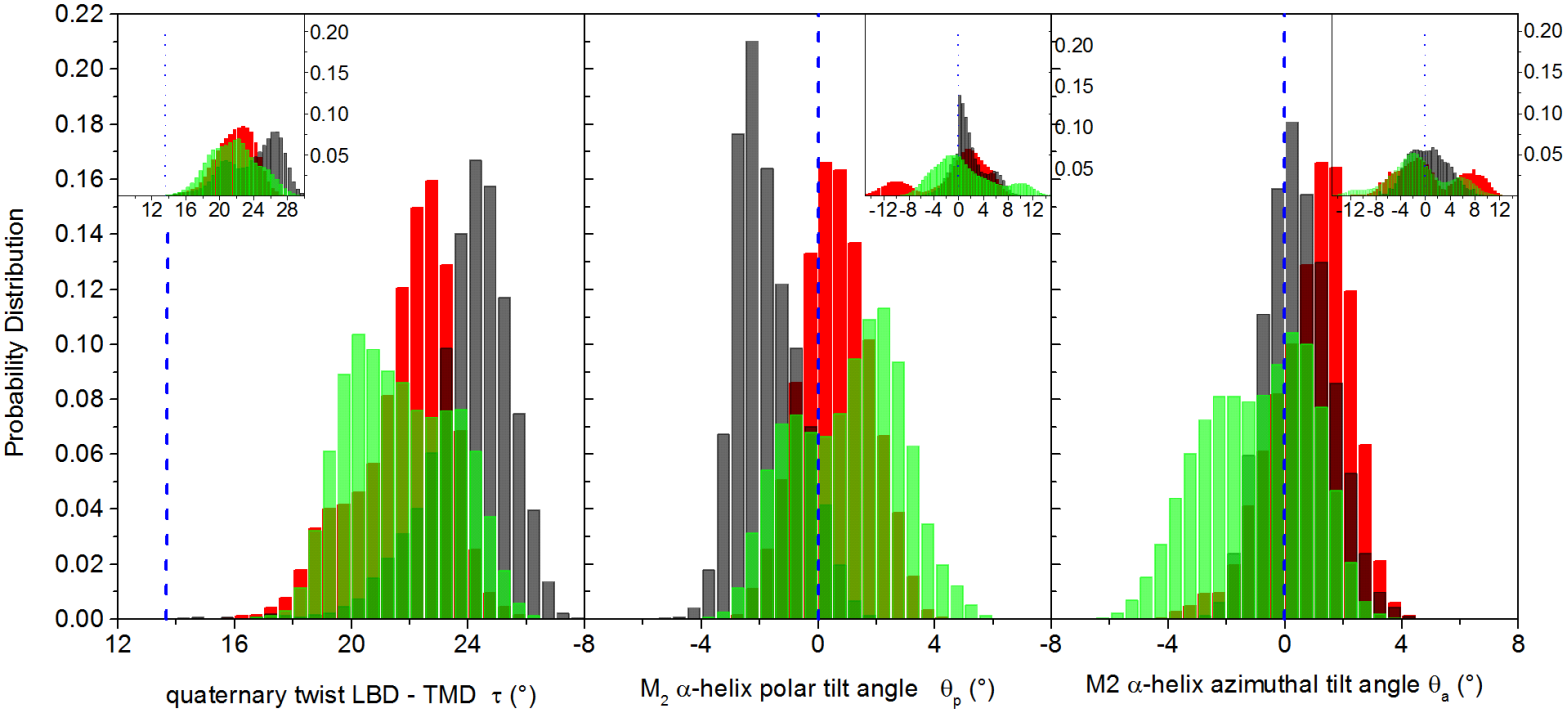
Twist and tilt angles. Left panel: distributions of quaternary twist angle; central panel: distributions of the M2 helix polar tilt angle; right panel: distributions of M2 helix azimuthal tilt angle. Values averaged over the five subunits. In the insets, distributions calculated collecting data from the five subunits. The vertical dashed blue line represents the value in the starting homology modeled configuration. Black bars: unrestrained; red bars: restrained; green bars: free simulations (data averaged over the four free trajectories).

Normal mode analysis on full-length models of nicotinic receptors pointed out that the lowest-frequency mode involves a global twisting motion of the LBD domain relative to the TMD domain, in which the domains rotate in opposite direction around the pore axis [47, 49, 85–87]. This conformational change has been related to the channel gating mechanism [47, 49]. The value of the twist angle gives a measure of gate locking when the channel is bound to antagonists or in the absence of ligands, and it is expected to be larger for a closed channel than for an open one. The distributions of the twist angle values in our trajectories are shown in Fig 9, left panel. Data are averaged over the five subunits. In the inset, the distributions of data collected from the five subunits are shown; this to highlight the contribution of individual subunits that would be otherwise blurred by simple averaging.

In agreement with the literature [53, 84], we observe in all trajectories a large change of the twist angle with respect to the homology model: the twist angle averaged over the trajectories relaxes to values higher than the one observed in the starting structure (Fig 9, left panel), but the protein undergoes a smaller twist along the restrained trajectory with respect to the unrestrained one, with average values of 21^°^ and and 24^°^, respectively.

Over the four free trajectories (distributions not shown), the twist angle is lower than in the restrained simulation in two cases (peak at ∼ 20^°^), similar to the restrained value in one case (∼ 21^°^) and close to the unrestrained value in the fourth case (∼ 24^°^). On average, as shown in Fig 9 (left panel, green bars), the twist angle is decreasing from the collapsed to the open conformations sampled both in the restrained and the free trajectories.

For sake of comparison, the value for simulated native GLIC and GluCl (in the presence of ivermectin) is about 15^°^, while in GluCl simulated in the absence of ivermectin the twist angle increase to 20^°^[53]. The value for the simulated closed ELIC structure is 23^°^. In a model of glycine receptor built on GLIC [84] and bound with ethanol to stabilize the open form, the twist angle is ∼ 14^°^, slightly smaller than the value of ∼ 16^°^ in the form obtained in the absence of ethanol. To summarize, the variation of twist observed in the present work agrees with the observations previously made in MD simulations: the twist angle increases with the tendency of the channel to close.

The orientation behavior of the M2 helices is more complex. The distributions of polar and azimuthal tilt angle components, calculated by averaging over the five subunits, are shown in Fig 9, central and right main panels, respectively. The distributions of data collected from the five subunits are also shown in the inset of each panel, highlighting the asymmetrical, non-concerted motion of the helices [10, 47, 48, 50, 88]. For the polar tilt, the central main panel shows a change from slightly negative values in the collapsed channel (black bars, peak at -2^°^) to slightly positive values (red bars, peak at 1.1^°^) in the putative open conformation obtained from the restrained simulation. This variation is in agreement with the channel pore shape going from a straight to a V-shaped form (Fig 4B and 4C). The values averaged over the four independent free trajectories have a distribution with higher weight for positive polar tilt angles. For sake of comparison, the polar tilt angle increases from ∼ 7^°^ in native GluCl simulated in the absence of ivermectin to ∼ 9^°^ in GluCl stabilized in the open form with ivermectin [53]. In simulations of prokaryotes [53], the polar tilt angle was ∼ 1^°^ in the closed channel of ELIC and ∼ 7^°^ in the GLIC open channel. As for the azimuthal tilt, in the restrained trajectory the distribution averaged over the five subunits (right main panel, red bars) shifts toward positive values with respect to the starting homology model, with a large variability among the five subunits (see inset). This high variability is even more evident in the four free trajectories. Typical values for the azimuthal angle found in other simulations [53] are -8.5^°^ in closed ELIC and ∼ 2^°^ in open GLIC; ∼ 3^°^ and ∼ 5^°^ in native GluCl in the absence and in the presence of ivemectin respectively. Overall, the slight increase of polar tilt angle from the closed to open conformations is in agreement with the observations previously made in the literature in X-ray structures and MD trajectories, although no specific values can be deciphered up to now.

### Ions dynamics

**Fig 10:**
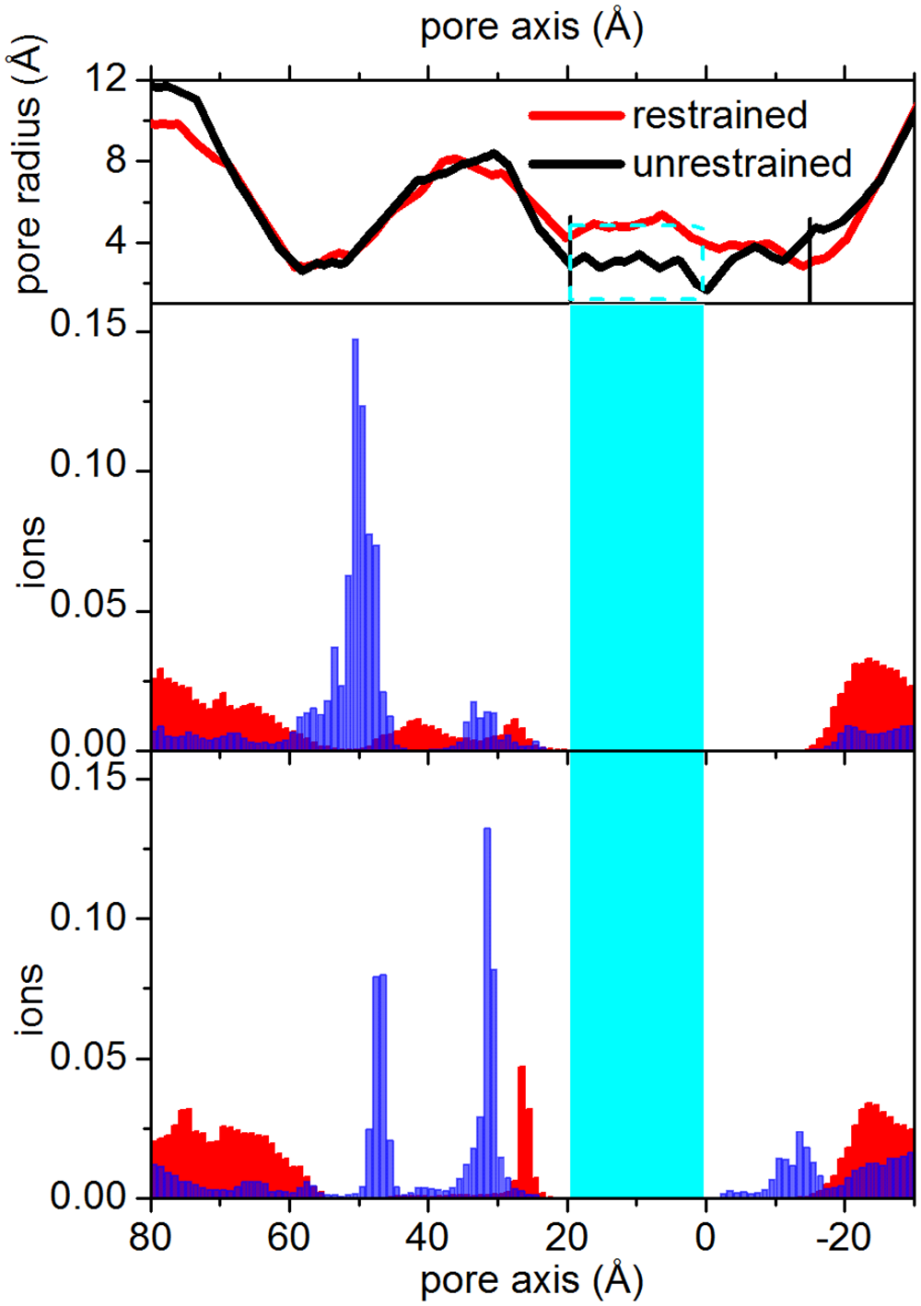
Ions distribution. Blue bars: cations, that can permeate the nicotinic channel. Red bars: anions. Top panels show the channel pore profiles along the full-length protein. Central panel: restrained; lower panel: unrestrained trajectory. The cyan region including the cyan rectangle in the top panel) highlights the channel between Glu20′ and Leu9′. The vertical black lines on the pore profile highlight the cytoplasmic and the extracellular pore limits, from Gly2′ to Glu20′.

The *α*7 nAChR is cation selective. To further assess the *α*7 model proposed in this study, we performed an analysis of the distribution of both cations and anions in the channel pore. Fig 10 shows the distribution of the z-coordinate of ions along the restrained and unrestrained trajectories, from the intracellular to the extracellular exits, both for the sodium (cations) and chlorine (anions) ions. Ion populations calculated along the free trajectories are shown in S9 Fig. The overall profile along the full-length channel is in agreement with results reported in literature for nicotinic receptors, though obtained with higher ions concentration and/or via the application of a transmembrane potential along Brownian Dynamics simulations (see e.g Fig 7 in Ref. [89]). Starting from the bottom of the protein (-30 Å), the anions populate more the cytoplasmic extremity of the channel (-30 Å<z<-20 Å), but the cations could enter more into the channel, in the portion comprised between -20Å and 0 Å; this in particular in the unrestrained trajectory and, on average, along the four free trajectories. The hydrophobic gate, in the range 0 Å<z<20 Å is not populated. Indeed, given the low, physiological salt concentration (100 mM) used here to neutralize the total system charge, and, most importantly, the time scale of our MD simulation (200ns for each trajectory), it is highly improbable to detect any conductive event crossing the hydrophobic gate, where the free energy barrier to ion permeation is located. However, despite the low concentration here used with respect to other studies devoted to ion motion [89, 90], along one of the free trajectories (Free_4_) we observed the simultaneous presence of two cations at the same time in the region just below the Leu9′ hydrophobic ring (-10 Å<z<0Å) [91]. This is a precursor event for the ion passage through the hydrophobic gate [89]. Ions population in the LBD shows an alternation of anions and cations. We observe in particular a cation excess located at z ∼ 30Å and z ∼ 50Å, in correspondence to charged residue rings composed by Asp38, Glu39 and Asp91, Glu92 respectively. These rings corresponds to the ones identified in Ref. [90] to be associated with prolonged cation dwell time.

### LBD-TMD interface

So far, we focused our attention mostly on the TMD portion of the protein. The above reported analyses of the pore structure, the hydration behavior, the M2 orientation and the ion dynamics point out that the structures we obtain, both in the restrained and free simulations, are representative of a stationary, active state of the channel. It is likewise important to assess how the remainder of the homology model we propose behave along the simulated trajectories.

**Fig 11:**
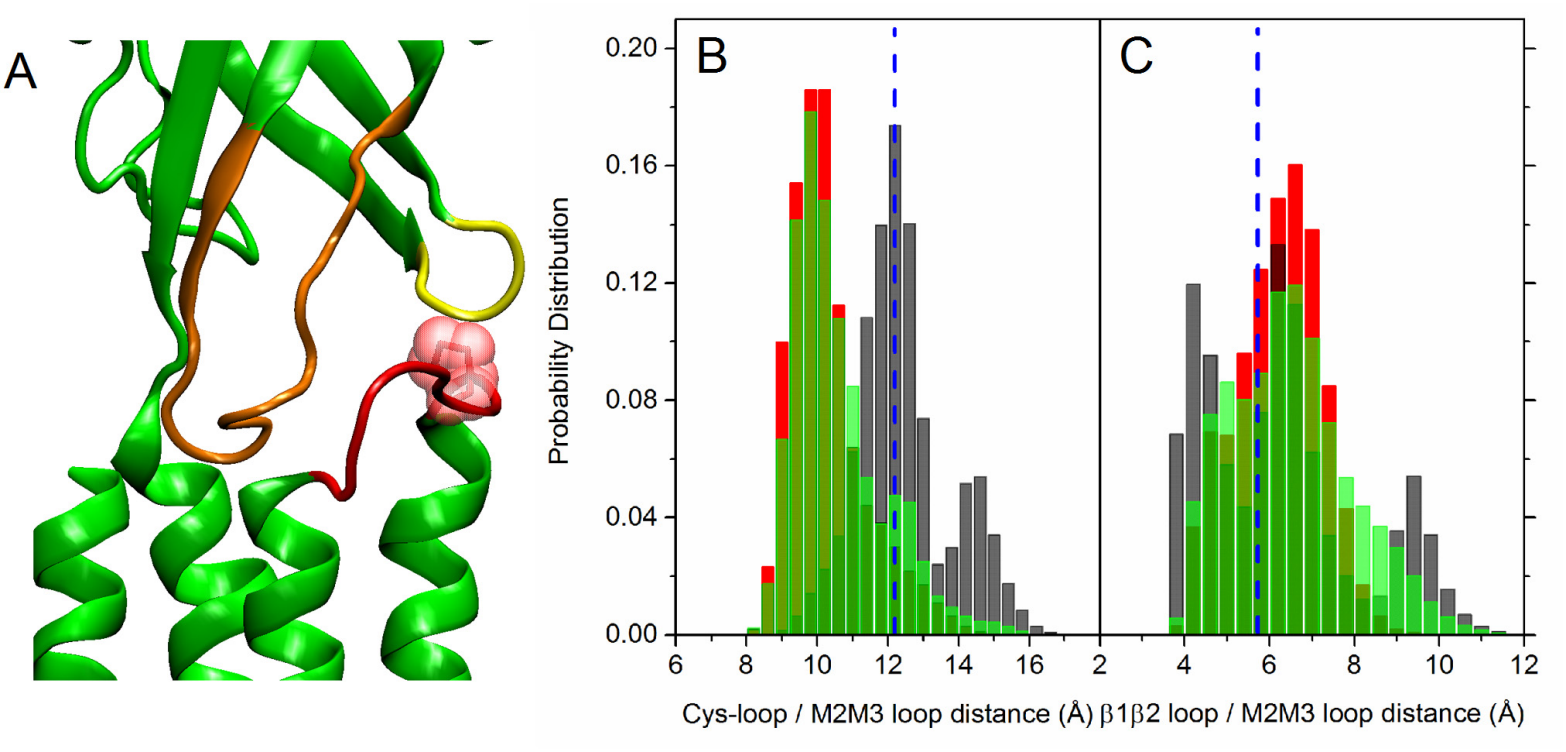
The LBD-TMD interface. Left panel: Cys-loop (orange), *β*1-*β*2 loop (yellow) and M2-M3 loop (red) in one subunit in the initial configuration. Pro256 belonging to the M2-M3 loop is also shown (red) in vdW representation. Central panel: distribution of the distances between the C_*α*_ atom of Pro256 on the M2-M3 loop and the center of mass of the Cys loop (*C*_*α*_ atoms of residues 122–136); right panel: distribution of the distances between the C_*α*_ atom of Pro256 on the M2-M3 loop and the C_*α*_ of residue Lys40 on the *β*1-*β*2 loop. Data collected from the five subunits. The vertical dashed blue line represents the value in the starting homology modeled configuration. Black bars: unrestrained; red bars: restrained; green bars: free simulations (data averaged over the four free trajectories).

The analysis of the nicotinic receptors gating mechanism pointed out that the TMD/LBD interface region is involved in the transmission of the signal from the LBD towards the channel region, through a cascade mechanism [8, 20, 46, 48]. Actually, the coupling of the agonist binding to channel gating has been explained on the basis of the concerted interaction of the M2-M3 loop in the TMD with the the Cys-loop and the *β*1-*β*2 loop in the LBD [56, 92] (Fig 11).

In particular, MD simulations have shown how the sliding motion of the M2-M3 loop from the *β*1-*β*2 loop towards the Cys-loop could be responsible for the initiation of pore widening in the closed-to-open transition in response to ligand binding [46, 48]. In the present work, we calculated the distance between i) the C*α* atom of Pro256 in the M2-M3 loop and the center of mass of the C*α* atoms in the Cys-loop (residues 122–136); ii) the C*α* atom of Pro256 and the C*α* atom of Lys40 in the *β*1-*β*2 loop. This choice has been done by careful comparison of our protein primary sequence with other models in the literature. Indeed, in the case of native GluCl [53], it was monitored just the distance between the C*α* of Pro268 on the M2-M3 loop and the C*α* of VAL45 on the *β*1-*β*2 loop; in the case of the *α*4*β*2 human nAChR modeled in Ref. [50], two distances were monitored, between the C*α* of Asp268 on the M2-M3 loop and the C*α* of Arg48 on the *β*1-*β*2 loop; between the C*α* of Lys274 on the M2-M3 loop and the C*α* of Asp140 on the Cys loop, respectively. Distributions of the distance values are shown in Fig 11, panel B and C respectively, for both the collapsed and open forms. In the latter case, the M2-M3 loop detaches from the *β*1-*β*2 loop (Fig 11C), and stably resides closer to the Cys-loop (Fig 11B). In good agreement with results for open forms reported in Refs. [50, 53], results point out that both the restrained and free simulations provide conformations representative of the full-length channel in an open form.

### Intra protein hydrogen bonds

A transition from a *tense* to a more *relaxed* form of the channel has been invoked recently [10] in the description of the closed-to-open mechanism of *Torpedo* nicotinic receptor; in particular it was proposed that, due to the release of interhelical contacts, the M2 helices become free to adopt a configuration more asymmetrical in the open form, with respect to the closed structure. In that respect, intra protein hydrogen bond analysis, performed along all MD trajectories, provided a quite interesting qualitative picture. A hydrogen bond is considered to be formed when the donor-acceptor distance is lower than 3.0Å. We detected many more hydrogen bonds along the unrestrained trajectory than in the restrained or free ones, both in the LBD and TMD. In particular two hydrogen bonds are found between the TMD M2-M3 loop and the *β*1-*β*2 loop in subunit P4; three hydrogen bonds between the M2-M3 loop in the subunit P1 and the *β*10 in the subunit P2; two hydrogen bonds between the M2-M3 loop in the subunit P1 and the Cys-loop in the subunit P2. Two other intersubunit hydrogen bonds are formed between the M3 helix in the subunit P3 and the Cys-loop in the subunit P4. Quite interestingly, four intra and one intersubunit hydrogen bond were detected involving the TMD helices: one hydrogen bond between the helices M1 and M3 in the subunits P1; three hydrogen bonds between the helices M2 and M4 in the subunit P3; and one hydrogen bond between the helix M1 in the subunit P5 and the helix M3 in the subunit P1. All the hydrogen bonds listed above are persistently formed in time (percentage >60%) in the collapsed structure, while totally absent or scarcely persistent (<5%) in the open structures. Accordingly, the collapsed structure results more tense than the open forms.

### C loops conformation and interaction with the agonist epibatidine

**Fig 12:**
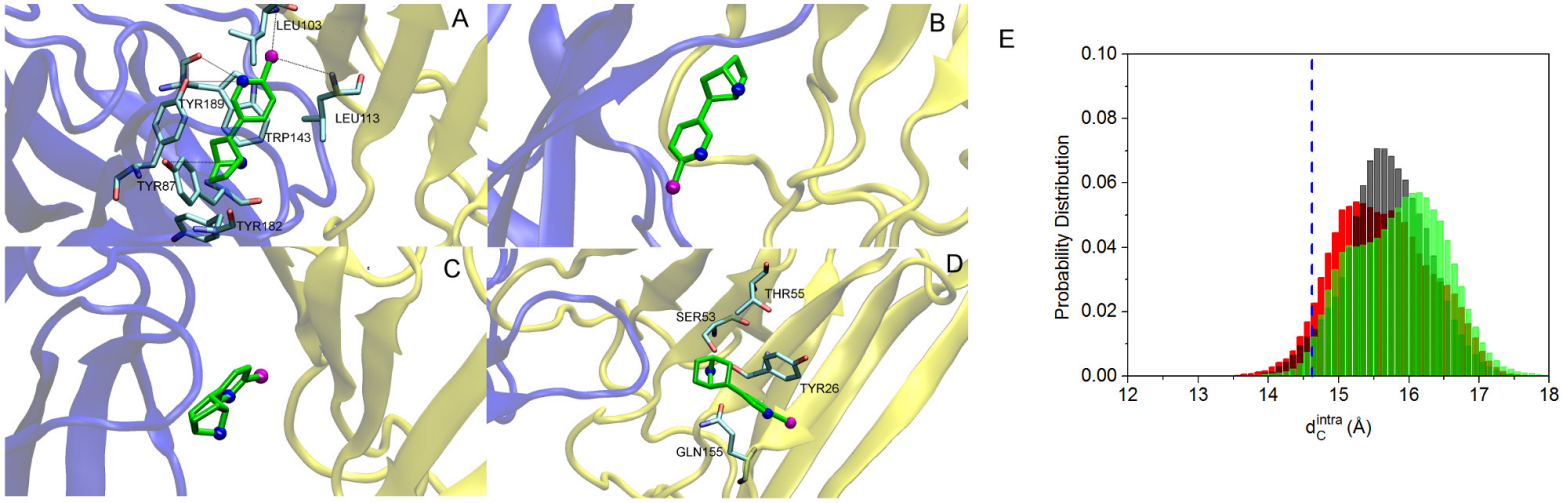
Protein-ligand interaction. A) Epibatidine and key residues (in licorice) in the binding pocket, in the homology model. The primary and complementary subunits are shown in blue and yellow cartoon representation, respectively. Epibatidine is in green. Chlorine (purple) and nitrogen (blue) atoms on epibatidine in ball representation. B) Representative snapshot along the Free_3_ trajectory with epibatidine in axial position, with the chlorine atom down; C) in equatorial position; D) epibaditine and key residues in the complementary subunit. E) distribution of $d_{C}^{intra}$. Data collected from the five subunits. The vertical dashed blue line represents the value in the starting homology modeled configuration. Black bars: unrestrained; red bars: restrained; green bars: free simulations (data averaged over the four free trajectories).

In nicotinic receptors the ligand pocket, present in each of the subunits, is delimited by the so-called loop C and extends at the interface between subunits. The ligand type influences the degree of C-loop closure against the protein core [25, 93, 94], as the loop arrangements cluster into three groups: i) the agonist-bound “open” conformation; ii) the apo “intermediate” conformations; iii) the antagonist-bound “closed” conformations. To further assess our *α*7 model we analyzed the ligand pocket by describing the behavior of the C loop capping the epibatidine molecules using the distance ($d_{C}^{intra}$) between the C loop center of mass and the center of mass of a couple of residues in the backwall. Protein-ligand interactions have been also monitored along the MD trajectories of the complexed system. According to crystallography [26], we selected and monitored three electrostatic interactions: the first between the epibatidine bridge ring amine and the Tyr87 side chain oxygen; the second between the epibatidine pyrimidine amine and the Tyr189 side chain oxygen; the third between the epibatidine pyrimidine amine and the Trp143 carbonyl oxygen. We also considered two other electrostatic interactions, those between the epibatidine aromatic chlorine and each of the two backbone nitrogens of Leu103 and Leu113 from the complementary subunit. Finally, we analyzed three van der Waals interactions: between the epibatidine pyrimidine ring and the Trp143 indole; between the epibatidine bridge ring and the Tyr189 aromatic ring; between the epibatidine bridge ring and the Tyr182 aromatic ring. The distance between the involved charged atom pairs has been calculated along the whole trajectories; the van der Waals interactions have been monitored through the distance between the center of mass of the bridge ring or of the pyrimidine ring and the center of mass of the residues aromatic rings. The epibatidine and the key residues above mentioned are shown in Fig 12, panel A. Distances averaged along the six trajectories are reported in Table 2. Results show that, on average, the ligand is kept in place during the restrained and free simulations.

**Table 2:** Protein-ligand distances.

Distance	Unrestrained	Restrained	Free_1_	Free_2_	Free_3_	Free_4_
EPI-N1/TRP143-O	6.2 ± 1.7	4.5 ± 0.9	4.7 ± 1.0	4.4 ± 0.9	5.1 ± 1.5	4.5 ± 0.9
EPI-N1/TYR189-OH	6.5 ± 2.4	5.3 ± 1.1	5.9 ± 1.4	4.9 ± 0.5	6.2 ± 2.1	5.3 ± 1.1
EPI-N2/TYR87-OH	6.2 ± 1.3	5.9 ± 2.0	6.0 ± 1.3	5.9 ± 1.2	6.6 ± 1.8	5.9 ± 2.0
EPI-CL/LEU103-N	8.9 ± 5.2	4.2 ± 0.8	4.1 ± 0.8	4.3 ± 0.8	5.4 ± 2.6	5.1 ± 1.2
EPI-CL/LEU113-N	8.2 ± 3.0	5.1 ± 1.2	5.3 ± 1.3	5.8 ± 1.2	6.5 ± 2.1	5.6 ± 1.6
EPI-pyrimidine/TRP143-indole	6.1 ± 1.3	5.0 ± 0.7	5.1 ± 0.8	5.3 ± 0.8	5.2 ± 0.9	4.9 ± 0.7
EPI-bridge ring/TYR189-ring	6.3 ± 2.5	5.3 ± 1.1	5.0 ± 0.7	4.9 ± 0.5	6.0 ± 2.2	5.5 ± 1.7
EPI-bridge ring/TYR182-ring	5.3 ± 0.7	5.2 ± 0.4	5.3 ± 0.4	5.4 ± 0.4	5.8 ± 1.2	5.6 ± 0.8

Distances (in Å) between epibatidine and selected residues (see Section “C loops conformation and interaction with the agonist epibatidine”). Values averaged along 200ns, using data collected from the five subunits.

The comparison with the unrestrained run suggests that the most of ligand/binding pocket interactions weaken if the channel collapses. This is confirmed for almost all subunits by an analysis of van der Waals and electrostatic interaction energies between the ligand and the pocket residues above mentioned (Table 3).

**Table 3:** Protein-ligand interaction energies.

Subunit	Trajectory	van der Waals	electrostatic
P1	Restrained	-15.7	-1.2
	Unrestrained	-10.9	-1.6
P2	Restrained	-13.6	-2.3
	Unrestrained	-11.8	-0.1
P3	Restrained	-14.9	-4.1
	Unrestrained	-13.7	-1.7
P4	Restrained	-12.8	-3.8
	Unrestrained	-18.6	-1.5
P5	Restrained	-12.3	-1.5
	Unrestrained	-11.2	-0.9

Interaction energies (in kcal/mol) between epibatidine and selected residues (see Section “C loops conformation and interaction with the agonist epibatidine”). Values averaged along the 100–200ns portion of the unrestrained or restrained trajectory. The error bar is within 3% in all cases.

As shown in Fig 12, panel E, the C-loop/backwall distance is, on average, 14.6 Å in the homology model; it increases to values between 15 Å and 16 Å in the open conformations. In particular, for the restrained trajectory the distribution shows peak maxima at 15.2, 15.9 and a shoulder at 16.5 Å; the open free trajectories provide a bimodal distribution, with peaks at 15.1 and 16.1 Å. This behavior corresponds to the different positions of the ligand in the binding pocket, as also observed in other bound ligand receptors [95, 96]. Indeed, in some subunits, the epibatidine undergoes several transitions from an axial to an equatorial orientation, i.e. pointing the chlorine atom up toward the apical side, as it was in the homology model (Fig 12, panel A), or flipped down (as in Fig 12, panel B) or radially toward the pore center (Fig 12, panel C). In the distributions of $d_{C}^{intra}$ the peaks at 15.2 and 15.1 Å are related to the ligand axial orientation, and the ones at 16.5 and 16.1 Å to the equatorial orientation. The shoulder at 15.9 Å in the restrained distribution is due to a position intermediate between the two. In one case, at the end of the free_3_ trajectory, the epibatidine detaches from the primary subunit, sliding toward the complementary subunit, where interactions with charged and aromatic side chains (e.g. Tyr26, Ser53, Thr55, Gln155) compete with the ones in the primary subunit to keep the ligand in place at the interface (Fig 12, panel D).

## Discussion

The knowledge of few high resolution crystal structures together with atomistic simulations, has recently started to yield valuable insight on the gating mechanism of nAChRs in response to ligand binding [13, 53–55, 97]. However, open-pore conformations of nAChRs are still scarcely available. In this respect, the X-ray structure determination of the prokaryotic pentameric GLIC channel, claimed in open form [15], has contributed to the understanding of the structure/dynamics/function relationships in nAChRs. However, stability assessment via full atomistic MD pointed out that GLIC undergoes a spontaneous closure [79, 97] and that pH variations are needed to stabilize the channel in the open form [11, 13]. In one study only, GLIC was simulated for 200ns without observing pore collapse [98].

With respect to the electron microscopy structures used in the past for modeling hetero- and homopentamers like the human *α*7, the X-ray structure of the glutamate receptor GluCl [41, 42] is undoubtedly a better template. Besides the higher atomic resolution, at variance with GLIC, GluCl is activated by ligands distinct from the solvent, and therefore much more suitable to study ligand-gated ion channels. Concerns have been raised however on the open channel stability of the X-ray structure of GluCL: recent, independent MD simulations [53–55] point out that the experimental open structure collapses towards a non-conductive, dehydrated channel; it has been shown that sustaining the open form is very difficult, already on the tens of nanoseconds time scale. This in principle could hamper its use for molecular modeling of the nicotinic receptors transition. Normal mode analysis has been used [55] in conjunction with an elastic network model of the protein to produce an open structure based on native GluCl; the putative open conformation was assessed along 100ns of MD. In this model, the M2 helices motion in the TMD controls the channel hydration, through an overall backbone and sidechain reorientation; the polar and azimuthal tilt angles were found to be 5.25^°^ and 1.0^°^, respectively. The radius of the obtained open pore was 5 Å.

The present work adds to the field, as, to the best of our knowledge, it is the first to use GluCl as template for a human nAChR. We created a chimeric model using the structure of GluCl [41] to model the TMD of the channel and the Cys-loop region, plus a known structure of AChBP to model the ECD of the channel bound to the agonist epibatidine [26]. The structure obtained can be considered as a model of the full-length human *α*7 pentamer in the active state both at the TMD and LBD level.

We have presented a combination of homology modeling and full-atomistic, explicit solvent MD simulations of the human nicotinic receptor *α*7. It is well known that the accuracy of a computational model is determined mostly by two factors [62]: i) the sequence identity between the target and the template, and ii) the quality of the template. Although the percentage of identity between our two sequences is low (25.37%), we obtained an optimal alignment of the functional regions and only few gaps. As for the quality of the template, we used crystallographic structures with the highest resolution available (2BYQ: 3.4 Å and 3RIF: 3.35 Å), as described in Materials and Methods. Unbiased MD in a lipid bilayer/water system was performed to assess the stability of the model and to refine it at high-resolution, in light of a well-established approach of combined static modeling and dynamical optimization [99–101]. Thus, our refinement is hampered only by the known limitations of MD simulations, namely the quality of the force field and the restricted conformational sampling [102]. Results show that the structure rapidly evolve towards a collapsed state, in agreement with the recent literature [53–55]. At variance with using normal modes to force the closed channel to assume the open form [55], in the present study we performed MD simulations by perturbing the chimeric model to maintain the channel pore open, using a restraint on the distances between M2 helices designed to switch on only when the helices tend to come close to each other. Remarkably, such local restraint on few specific, functional residues let us observe the spontaneous change of other, not directly affected regions towards the stable structure with a wide pore.

Overall, such structure was preserved for a cumulative simulation time of about 1 *μ*s, 800ns of which are unbiased, free trajectories. Based mostly on the analysis of the level of hydration, pore size and profile (see Fig 8 and S8 Fig) and supported from the comparison with other putative open forms reported in the literature [15, 50, 55], we suggest that the obtained structure has reasonable chances to be (at least one of the possible) structures of the channel in the open conformation.

An unsolved issue about channel receptors concerns the different states of the receptor described by the electro-physiological experiments: open, closed, resting, desensitized. There is no clear correspondence between these experimental observations and simple structural criteria, beside the C loop opening/closure in the LBD. This structural feature was considered in the past as related to the type of ligand, but this is in contradiction with recent results on a AChBP structure bound to dihydro-beta-erythroidine (DH*β*E)[103], which is an antagonist, but so small that the C loop does not open more than in presence of an agonist.

It is known that the M2 helices play a role in channel gating but parameters describing rigid-body motions of the M2 helices such as helices tilting, or the quaternary LBD/TMD twist, are not sufficient to describe the complex motions in the pore. Discerning open from closed structures based on these structural quantities can be an ambiguous task, as already observed for both heteropentameric [50] and homopentameric [46] models of human nAChRs. In particular, it has been reported that the M2 helices undergo a non- concerted motion, with no correlation between the radial and lateral tilting [50], in which channel opening is driven by the changes in the sidechain orientations of few hydrophobic pore-lining residues, similarly to what we find in this work. In this respect, the analysis of the pore profile and hydration is much more informative. Our *α*7 nAChR we model has a pore consistent with an open channel, slightly larger than the pore in the open model of *α*4*β*2 nAChR [50], the modeled system closest to the one modeled here. Furthermore, the comparison with hydrophobic pore models and GLIC [83, 88] points out that the stationary hydration level in our putative open structure is consistent with the one of a conductive channel. Atomic level details of the TMD-LBD interface, the protein hydrogen bonds network, and the epibatidine conformations at the binding sites contribute to and support the characterization of the active channel both in the extracellular and transmembrane regions.

To conclude, this work provides a first step towards obtaining a reliable and stable all-atom model of the channel in an open form. Recently it has be observed the existence of multiple conformations for a given state, in GLIC [104], Torpedo nAChR [10] and in ELIC [105]. In this respect, the structure we found could be considered a good candidate for a distribution of conformations adequate to the functional open state. Our results potentially open multiple lines of investigation; indeed, the new structure could be used for studying the nAChR gating transition in path-sampling simulations, where the reliability of end-point structures is a fundamental requirement [106–108], or in virtual screening studies to dock different ligands.

## Supporting Information

S1 Fig The epibatidine. Schematic structure of the epibatidine molecule.
(TIF)

S2 Fig Crossed distances. Time evolution of the crossed distances between Val13′ (left panels) and Leu16′ (right panels) centers of mass, in facing subunits, for each pair of subunits, along the restrained simulations. The horizontal red lines mark the values calculated on the initial structure, i.e. the threshold values used in the implemented flat-bottom quadratic restraint.
(TIF)

S3 FigStability assessment.Left panels: Root Mean Square Deviation (in Å) of individual subunits calculated from the starting conformations (after equilibration) as $RMSD(t_{j})=\sqrt{\frac{\overset{N_{\alpha }}{\underset{C_{\alpha }=1}{\sum }}{\left(r_{C_{\alpha }}(t_{j})-r_{C_{\alpha }}^{ref}\right)}^{2}}{N_{\alpha }}}$, where **r**_*C*_*α*__(*t*_*j*_) is the position of the *C*_*α*_ atom at *j*th time step, ${\text{r}}_{C_{\alpha }}^{ref}$ is the position of the *C*_*α*_ atom in the reference structure, *N*_*α*_ is the total number of *C*_*α*_ atoms in the subunit. The RMSD values are calculated after removing the roto-translational body motions of the single subunits [109]. Upper row panels: unrestrained; lower row panels: restrained trajectory. Right panels: Root Mean Square Fluctuations (RMSFs) (in Å) of *C*_*α*_ carbon atoms in the individual subunits with respect to the average structure calculated along the final 100ns of the trajectories. The curves are colored according to the scheme in the inset. The bold black line in the RMSFs plot is the average over the five subunits. Upper row panels: unrestrained; lower row panels: restrained trajectory.(TIF)

S4 Fig Stability assessment. Left panels: *C*_*α*_ atoms Root Mean Square Deviation (in Å) calculated from the corresponding starting conformation after removing the roto-translational body motions [109], along the four free trajectories. Right panels: Root Mean Square Fluctuations (RMSFs) (in Å) of the protein *C*_*α*_ atoms with respect to the average structure calculated along the final 100ns of the trajectory, for the four free trajectories. Both RMSD and RMSFs values are averaged over the five subunits.
(TIFF)

S5 Fig Crossed distances. Time evolution of the crossed distances between Val13′ (upper panel) and Leu16′ (lower panel) *C*_*α*_ atoms in facing subunits, for each pair of subunits, in the first 20ns of MD including the equilibration stage. Solid lines: along the restrained simulation; dotted line: along the unrestrained simulation. The curves are colored according to the scheme in the inset in S3 Fig.
(TIF)

S6 Fig Hydration behavior. Top panel: time evolution of water count in the pore lumen delimited by the M2 helices. Bottom panel: time evolution of water count in a region of 10 Å centered at the constriction point 13′ (Val246). Black curve: unrestrained; red curve: restrained trajectory. Note dewetting-rewetting transitions occurring along the trajectory Free_3_ (violet curve) at ∼ 115ns, ∼ 135ns, ∼ 145ns, ∼ 180ns.
(TIF)

S7 Fig Hydration behavior. Representative snapshots of water molecule arrangements inside the pore at two different times along the Free_4_ trajectory (in the portion 145–150ns). Upper row: water molecules within 3Å from any atom of Ser10′ residues. Lower row: water molecules within 3Å from any atom of Thr6′ residues. All Ser10′ and Thr6′ residues are in vdW representation, colored in cyan.
(TIFF)

S8 Fig Pore profiles. Pore radius profiles along the pore region in configurations averaged over the last 100ns of the four free MD trajectories. Also reported the profile for the collapsed channel. Pore radius computed with MolAxis [80].
(TIF)

S9 Fig Ion distributions. Ion distributions in the free trajectories. Blue bars: cations, that can permeate the nicotinic channel; red bars: anions. Top panels show the channel pore profiles along the full-length protein. The cyan region highlights the channel between Glu20′ and Leu9′.
(TIF)

S1 Text Epibatidine parameters. Topology and epibatidine partial atomic charges.
(PDF)
