# Supplementary figures and images for: A Structural Model of the Human α7 Nicotinic Receptor in an Open Conformation
Source: PLoS One. 2015 Jul 24;10(7):e0133011. doi: 10.1371/journal.pone.0133011 (PMC4514475; doi:10.1371/journal.pone.0133011)

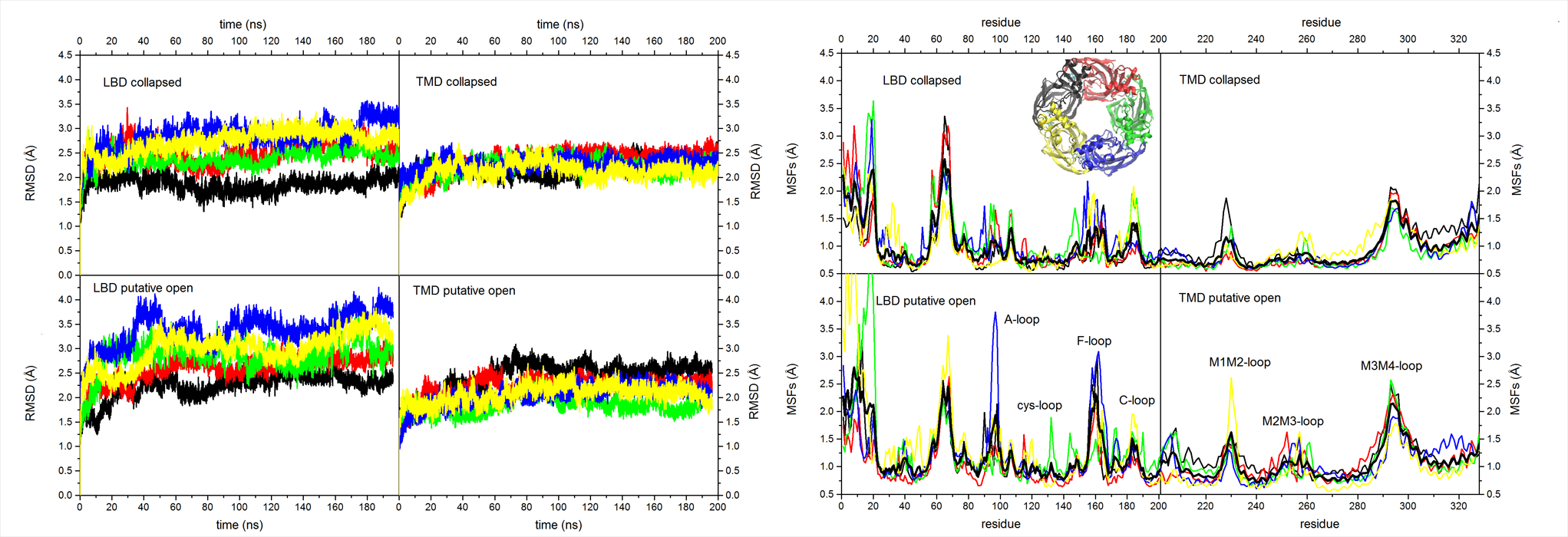

Supplement: S3 Fig — Left panels: Root Mean Square Deviation (in Å) of individual subunits calculated from the starting conformations (after equilibration) as RMSD(tj)=∑Cα=1Nα(rCα(tj)−rCαref)2Nα, where r Cα(t j) is the position of the C α atom at jth time step, rCαref is the position of the C α atom in the reference structure, N α is the total number of C α atoms in the subunit. The RMSD values are calculated after removing the roto-translational body motions of the single subunits [109]. Upper row panels: unrestrained; lower row panels: restrained trajectory. Right panels: Root Mean Square Fluctuations (RMSFs) (in Å) of C α carbon atoms in the individual subunits with respect to the average structure calculated along the final 100ns of the trajectories. The curves are colored according to the scheme in the inset. The bold black line in the RMSFs plot is the average over the five subunits. Upper row panels: unrestrained; lower row panels: restrained trajectory. (TIF) [file pone.0133011.s003.tif]

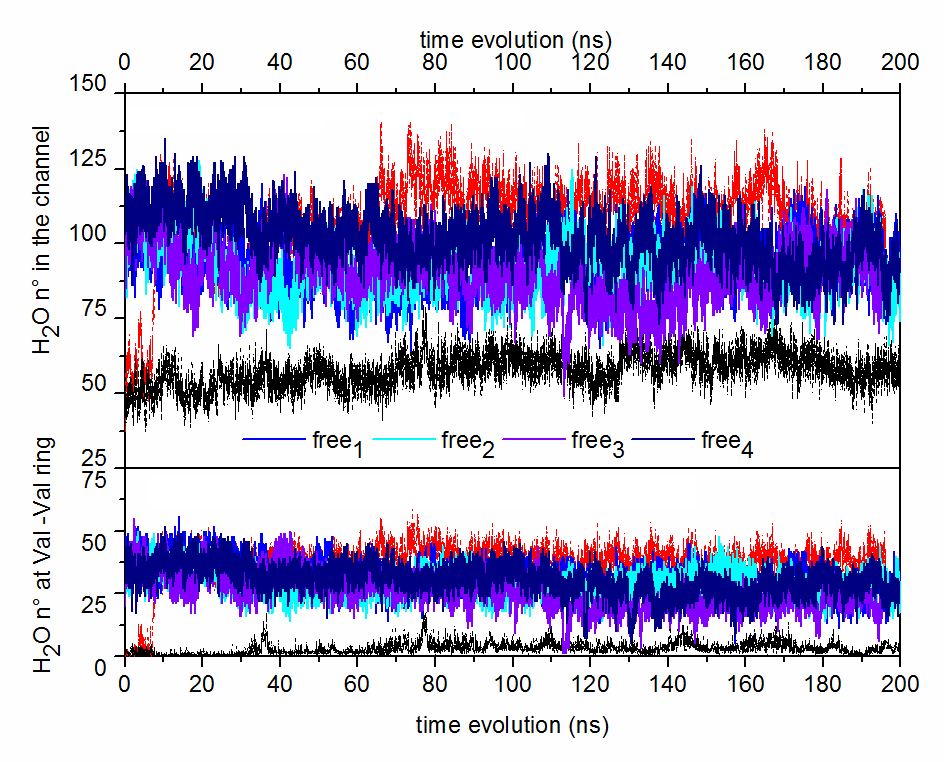

Supplement: S6 Fig — Top panel: time evolution of water count in the pore lumen delimited by the M2 helices. Bottom panel: time evolution of water count in a region of 10 Å centered at the constriction point 13′ (Val246). Black curve: unrestrained; red curve: restrained trajectory. Note dewetting-rewetting transitions occurring along the trajectory Free3 (violet curve) at ∼ 115ns, ∼ 135ns, ∼ 145ns, ∼ 180ns. (TIF) [file pone.0133011.s006.tif]

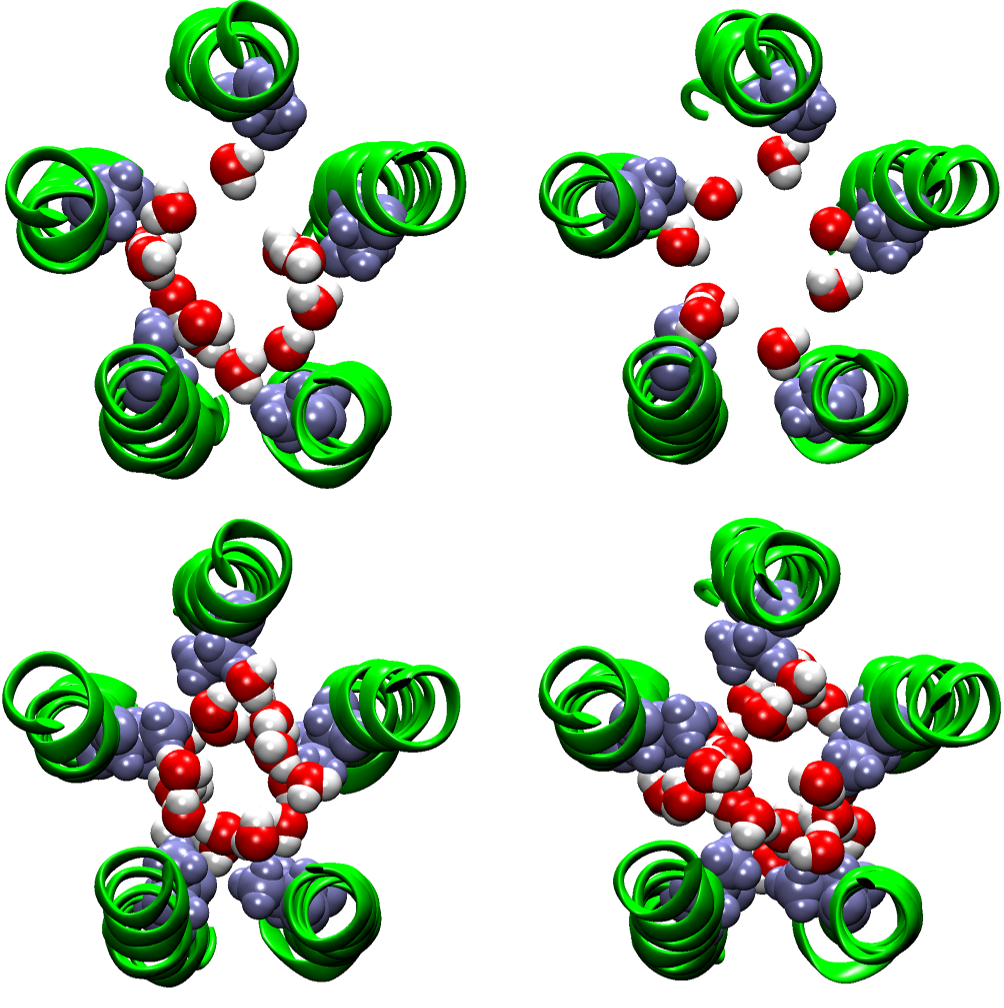

Supplement: S7 Fig — Representative snapshots of water molecule arrangements inside the pore at two different times along the Free4 trajectory (in the portion 145–150ns). Upper row: water molecules within 3Å from any atom of Ser10′ residues. Lower row: water molecules within 3Å from any atom of Thr6′ residues. All Ser10′ and Thr6′ residues are in vdW representation, colored in cyan. (TIFF) [file pone.0133011.s007.tiff]
