# Supplementary material for: A Structural Model of the Human α7 Nicotinic Receptor in an Open Conformation
Source: PLoS One. 2015 Jul 24;10(7):e0133011. doi: 10.1371/journal.pone.0133011 (PMC4514475; doi:10.1371/journal.pone.0133011)
Supplement: S1 Text — Topology and epibatidine partial atomic charges. (PDF) [file pone.0133011.s010.pdf]

EPIBATIDINE TOPOLOGY AND FORCE FIELD PARAMETERS

MASS	1	CAEP	12.010000
MASS	2	NBEP	14.010000
MASS	3	C3EP	12.010000
MASS	4	N3EP	14.010000
MASS	5	CLEP	35.450000
MASS	6	HAEP	1.008000
MASS	7	H4EP	1.008000
MASS	8	HCEP	1.008000
MASS	9	H1EP	1.008000
MASS	10	HNEP	1.008000

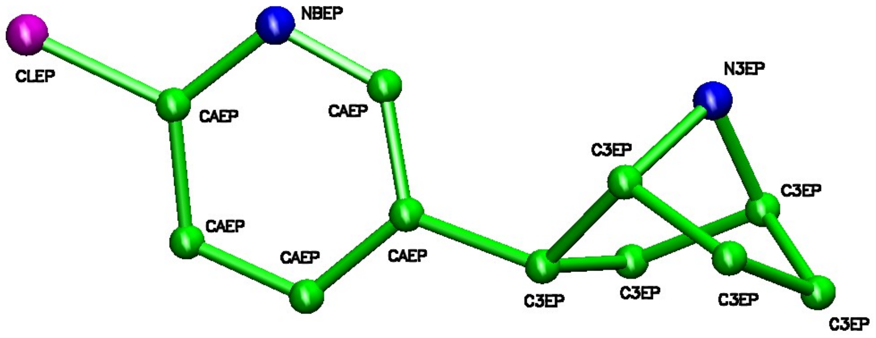

RESI EPJ 0.000
GROUP

ATOM C1	CAEP	0.622911
ATOM N1	NBEP	-0.627378
ATOM C2	CAEP	0.445547
ATOM C3	CAEP	-0.361159
ATOM C4	CAEP	0.109501
ATOM C5	CAEP	-0.379982
ATOM C6	C3EP	0.103899
ATOM C7	C3EP	-0.198116
ATOM C8	C3EP	0.213445
ATOM N2	N3EP	-0.597024
ATOM C9	C3EP	-0.025018
ATOM C10	C3EP	0.039794
ATOM C11	C3EP	-0.182912
ATOM Cl1	CLEP	-0.144477
ATOM H1	HAEP	0.152884
ATOM H2	HAEP	0.085448
ATOM H3	H4EP	-0.045533
ATOM H4	HCEP	0.077382
ATOM H5	HCEP	0.070098
ATOM H6	H1EP	0.039502
ATOM H7	HCEP	0.070098
ATOM H8	HCEP	0.050828
ATOM H9	HCEP	0.050828
ATOM H10	HCEP	0.008790
ATOM H11	HCEP	0.008790
ATOM H12	H1EP	0.083825
ATOM H13	HNEP	0.328031

BONDS

CAEP NBEP	483.10	1.342
CAEP CAEP	478.40	1.387
CAEP H4EP	342.90	1.088
CAEP CLEP	322.80	1.729
CAEP HAEP	344.30	1.087
C3EP CAEP	323.50	1.513
C3EP C3EP	303.10	1.535
C3EP HCEP	337.30	1.092
C3EP N3EP	320.60	1.470
C3EP H1EP	335.90	1.093
HNEP N3EP	394.10	1.018

ANGLES

CAEP NBEP CAEP	68.590	115.860
CAEP CAEP CAEP	67.180	119.970
CAEP CAEP C3EP	63.840	120.630
NBEP CAEP CAEP	69.160	122.630
NBEP CAEP H4EP	51.820	115.940
NBEP CAEP CLEP	65.810	116.150
CAEP CAEP HAEP	48.460	120.010
CAEP CAEP CLEP	62.920	119.400
CAEP CAEP H4EP	48.240	121.090
CAEP C3EP C3EP	63.250	112.090
CAEP C3EP HCEP	46.960	110.150
C3EP C3EP C3EP	63.210	110.630
C3EP C3EP HCEP	46.370	110.050
C3EP C3EP N3EP	66.180	110.380
C3EP C3EP H1EP	46.360	110.070
C3EP N3EP C3EP	64.010	110.900
C3EP N3EP HNEP	47.130	109.920
N3EP C3EP H1EP	49.390	109.920
HCEP C3EP HCEP	39.430	108.350

DIHEDRALS

X CAEP NBEP X	4.800	2	180.0
X CAEP CAEP X	3.625	2	180.0
X C3EP CAEP X	0.000	2	0.0
X C3EP C3EP X	0.156	3	0.0
C3EP C3EP C3EP C3EP	0.180	3	0.0
C3EP C3EP C3EP C3EP	0.250	2	180.0
C3EP C3EP C3EP C3EP	0.200	1	180.0
HCEP C3EP C3EP C3EP	0.160	3	0.0
HCEP C3EP C3EP HCEP	0.150	3	0.0
X C3EP N3EP X	0.300	3	0.0
C3EP C3EP C3EP C3EP	0.300	3	0.0
C3EP C3EP N3EP C3EP	0.480	2	180.0

IMPROPER DIHEDRALS

CAEP H4EP CAEP NBEP	1.100	2	180.0
CAEP CLEP CAEP NBEP	1.100	2	180.0
X X CAEP HAEP	1.100	2	180.0
C3EP CAEP CAEP CAEP	1.100	2	180.0

NONBONDED

!		Emin	Rmin/2		Emin/2	Rmin (for 1-4's)
!	(kCALOl/mol)		(A)			
CAEP	0.00	-0.0860	1.9080	0.00	-0.0430	1.9080
NBEP	0.00	-0.1700	1.8240	0.00	-0.0850	1.8240
C3EP	0.00	-0.1094	1.9080	0.00	-0.0547	1.9080
N3EP	0.00	-0.1700	1.8240	0.00	-0.0850	1.8240
CLEP	0.00	-0.2650	1.9480	0.00	-0.1325	1.9480
HAEP	0.00	-0.0150	1.4590	0.00	-0.0075	1.4590
H4EP	0.00	-0.0150	1.4090	0.00	-0.0075	1.4090
HCEP	0.00	-0.0157	1.4870	0.00	-0.0078	1.4870
H1EP	0.00	-0.0157	1.3870	0.00	-0.0078	1.3870
HNEP	0.00	-0.0157	0.6000	0.00	-0.0078	0.6000
